# The multifunctional polydnavirus TnBVANK1 protein: impact on host apoptotic pathway

**DOI:** 10.1038/s41598-017-11939-x

**Published:** 2017-09-18

**Authors:** Rosanna Salvia, Gerarda Grossi, Angela Amoresano, Carmen Scieuzo, Marisa Nardiello, Chiara Giangrande, Ilaria Laurenzana, Vitalba Ruggieri, Sabino A. Bufo, S. Bradleigh Vinson, Monica Carmosino, David Neunemann, Heiko Vogel, Pietro Pucci, Patrizia Falabella

**Affiliations:** 1 Department of Sciences, University of Basilicata, Via dell’Ateneo Lucano 10, 85100 Potenza, Italy; 2 Department of Chemical Sciences, Federico II University, Via Cintia 6, 80126 Naples, Italy; 3 Laboratory of Preclinical and Translational Research, IRCCS Referral Cancer Center of Basilicata (CROB), Via Padre Pio 1, 85028 Rionero in Vulture (PZ), Italy; 4 Department of Entomology, Texas A&M University, 370 Olsen Blvd, College Station, TX 77843-2475 USA; 5 Department of Entomology, Max Planck Institute for Chemical Ecology, Hans-Knöll-Straße 8, D-07745 Jena, Germany

## Abstract

*Toxoneuron nigriceps* (Hymenoptera, Braconidae) is an endophagous parasitoid of the larval stages of the tobacco budworm, *Heliothis virescens* (Lepidoptera, Noctuidae). The bracovirus associated with this wasp (*Tn*BV) is currently being studied. Several genes expressed in parasitised host larvae have been isolated and their possible roles partly elucidated. *Tn*BVank1 encodes an ankyrin motif protein similar to insect and mammalian IκB, an inhibitor of the transcription nuclear factor κB (NF-κB). Here we show that, when *Tn*BVank1 was stably expressed in polyclonal *Drosophila* S2 cells, apoptosis is induced. Furthermore, we observed the same effects in haemocytes of *H*. *virescens* larvae, after *Tn*BVank1 *in vivo* transient transfection, and in haemocytes of parasitised larvae. Coimmunoprecipitation experiments showed that *Tn*BVANK1 binds to ALG-2 interacting protein X (Alix/AIP1), an interactor of apoptosis-linked gene protein 2 (ALG-2). Using double-immunofluorescence labeling, we observed the potential colocalization of *Tn*BVANK1 and Alix proteins in the cytoplasm of polyclonal S2 cells. When Alix was silenced by RNA interference, *Tn*BVANK1 was no longer able to cause apoptosis in both S2 cells and *H*. *virescens* haemocytes. Collectively, these results indicate that *Tn*BVANK1 induces apoptosis by interacting with Alix, suggesting a role of *Tn*BVANK1 in the suppression of host immune response observed after parasitisation by *T*. *nigriceps*.

## Introduction

Endophagous parasitoids belonging to the order of Hymenoptera develop during juvenile stages in the body of their hosts. During oviposition, the endoparasitoid female injects into the host several factors that alter its physiology, suppressing the host immune system, the endocrine balance, as well as reproductive activity or metabolism^1^. Among parasitoid secretions, viruses belonging to the family of *Polydnaviridae* (PDVs) play a key role in the success of parasitism^2,3^. PDVs are obligate symbionts of ichneumoid and braconid wasps attacking exclusively larval stages of their lepidopteran hosts^4^. Polydnavirus DNA is integrated into the genome of the parasitoid and therefore vertically transmitted through the germline^4^. The viral particles, containing circular dsDNA molecules of different sizes, replicate only in the epithelium of parasitoid female ovarian calyx, and are injected into the host, along with ovarian proteins, venom and the egg, during oviposition. PDVs express genes in several target tissues of the host and, together with other maternal or embryonic factors, lead to several functional alterations of their host, providing a suitable nutritional environment to the parasitoid larvae^5^. *Toxoneuron nigriceps* (Viereck) (Hymenoptera, Braconidae) is an endoparasitoid wasp belonging to the Braconidae family, parasitising the larval stages of tobacco budworm *Heliothis virescens* (Fabricius) (Lepidoptera, Noctuidae). *Toxoneuron nigriceps* Bracovirus (*Tn*BV) encodes several gene families^6,7^ including ankyrin motif proteins (ANKs)^8^. The *Tn*BVANK proteins show high levels of amino acid identity to the ankyrin motif of the *Drosophila* NF-κB inhibitor (IκB)-related protein cactus^8,9^. Three ankyrin-like open reading frames (ORFs) were identified in the *Tn*BV genome and were denoted *Tn*BVank1-3^8^. *Tn*BVank genes are characterised by ankyrin motif sequence repeats but the usually phosphorylatable serine residues at the N-terminus are absent^8^. Moreover, none of putative proteins encoded by the *Tn*BVank genes has the C-terminal PEST domain present in the protein Cactus/IκB, which is necessary for signal-mediated degradation^10–12^ and for the control of protein turnover^13^. The predicted structure of these proteins suggested that *Tn*BV ankyrin proteins may bind NF-kB/Rel transcription factors of the tumour necrosis factor (TNF)/Toll immune pathway altering the signal transduction cascade^8^. Indeed, it was demonstrated that in parasitised *H*. *virescens* larvae, after bacterial challenge, the nuclear import of NF-κB was inhibited^8^. Moreover, transfection experiments in human HeLa cells demonstrated that a *Tn*BVank1 gene product reduced the efficiency of the TNF-α-induced expression of a reporter gene under NF-κB transcriptional control, confirming that viral IκB*-*like proteins are involved in the suppression of the insect immune response^8,14,15^.

*Tn*BVank1 and *Tn*BVank3 are expressed as early as three hours and six hours, respectively, after parasitisation in haemocytes of *H*. *virescens* last instar larvae, but the transcripts are present also several hours after parasitisation reducing over the time. Although expression of *Tn*BVank1 and *Tn*BVank3 is high in *H*. *virescens* haemocytes, a much more limited expression level can also be found in the fat body^8^. Moreover, it is reported that several hours after natural parasitism, the granulocytes, a subpopulation of haemocytes, showed a number of structural damages, as evidenced by actin cytoskeleton disruption and loss of adhesion properties, with general morphological changes which suggest the occurrence of apoptosis^16^. This further suggests a possible role of parasitism in early immune response suppression.

In insects the processes of cell death are involved in development, but also play an important role in allowing, maintaining and regulating many host-parasitoid interactions. Accordingly, it was shown that parasitoids are able to induce or regulate cell death phenomena in host tissues^16–20^. Here, we report that *Tn*BVANK1 induces cell death in polyclonal *Drosophila* S2 cells that stably express *Tn*BVank1. To better understand the role of *Tn*BVANK1, coimmunoprecipitation and coimmunolocalisation experiments were carried out in polyclonal *Drosophila* S2 cells and the interaction between *Tn*BVANK1 and ALG-2 interacting protein X (Alix) was investigated. Alix is a multifunctional protein, it is ubiquitously expressed and concentrated in phagosomes and exosomes and it was first characterised as an interactor of apoptosis-linked gene protein 2 (ALG-2) that is a Ca^2+^-binding protein necessary for cell death^21^. Here, we show that upon Alix silencing by RNA interference (RNAi), *Tn*BVANK1 was no longer able to induce apoptosis in S2 cells.

These data were further confirmed by *in vivo* transient transfection of *Tn*BVank1 and by silencing of Alix transcript by RNAi in *H*. *virescens* larval haemocytes.

## Results

### Expression of *Tn*BVank1 in S2 cells induces apoptosis

**Figure 1:**
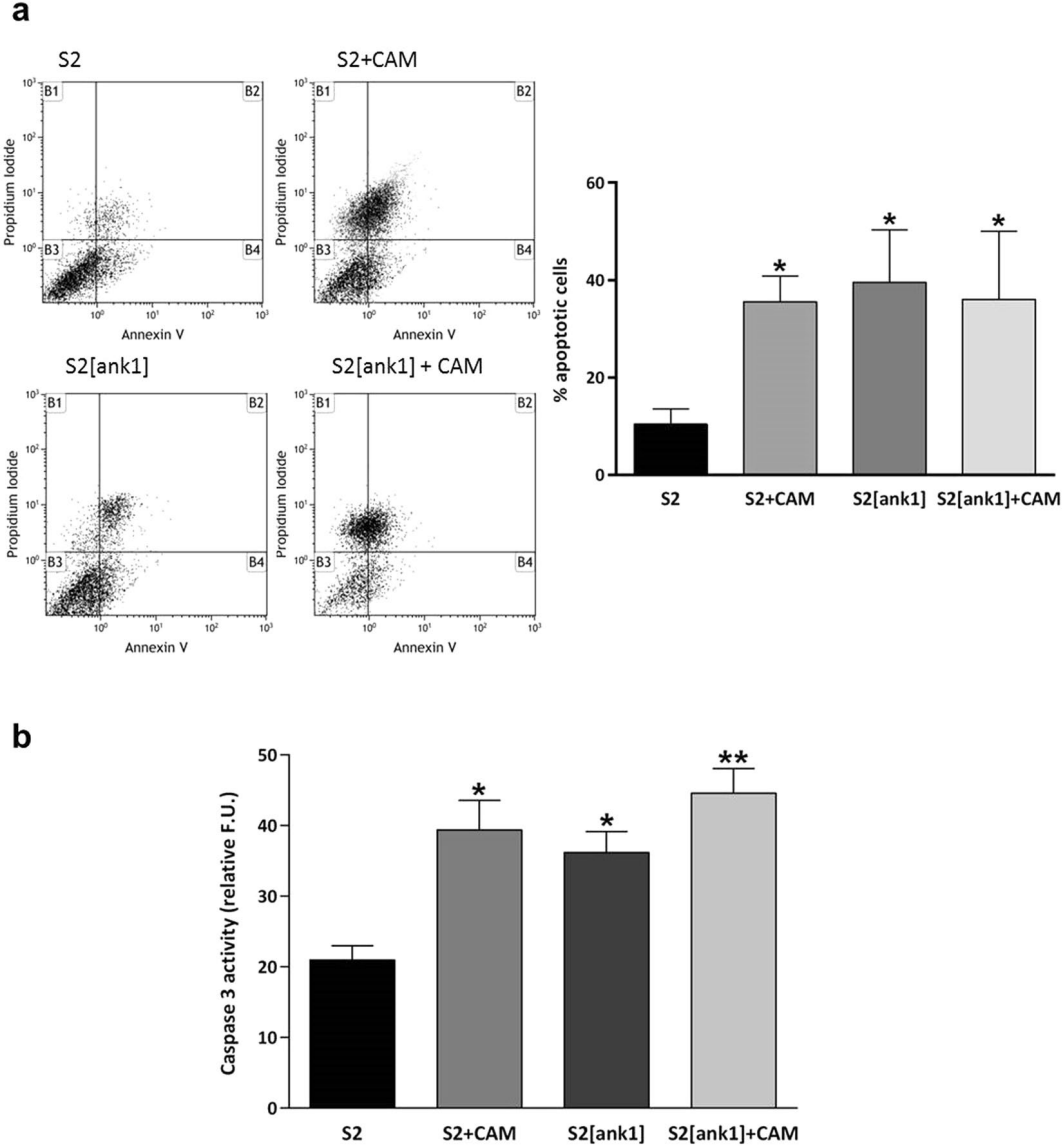
*Tn*BVank1 induces apoptosis and stimulates caspase-3 activity in S2 cells stably expressing *Tn*BVank1 gene (S2[ank1]). Apoptosis was induced in positive control S2 cells and S2 cells stably expressing *Tn*BVank1 through a treatment with 1 µM camptothecin (S2 + CAM, S2[ank1] + CAM). (**a**) Cells were harvested and doubly labeled with Annexin V-FITC and PI before analysis by flow cytometry. Dot plots of single positive for Annexin V and double positive for Annexin V and PI cells were interpreted as signs of early and late phases of apoptosis respectively (lower and upper right quadrant respectively). Data are quantified in the bar chart on the right showing the percentage of apoptotic cells. (**b**) Caspase-3 activity was determined using Ac-DEVD-AMC as a fluorescent substrate. For the assay, 3 × 10^5^ cells were used. Results are expressed as fluorescence units (F.U.); an excitation wavelength of 380 nm and an emission wavelength of 440 nm were utilised. Data represent the mean of 3 replicates ± SD. Statistically significant differences are indicated with asterisks (*P ≤ 0.05, **P ≤ 0.01, one way ANOVA and Tukey’s Test P < 0.05).

We analysed the profile of polyclonal S2 cells stably expressing *Tn*BVank1 (S2[ank1]) by flow cytometry. We used S2 cells and polyclonal S2[ank1] cells 20 h after treatment with the cytotoxic alkaloid camptothecin as positive controls and untransfected S2 cells as negative control. All the cell samples were harvested at the same splitting passages and were doubly stained with Annexin V–FITC and PI (Propidium Iodide). Results revealed a significant increase of apoptotic cells in polyclonal S2[ank1] cells line compared to the negative control, indeed the percentage of apoptotic cells, amounted to 39.56%, was not significantly different from the positive controls (S2 + CAM and S2[ank1] + CAM) (Fig. 1a).

To further investigate the involvement of *Tn*BVank1 in cell death pathway, we analysed also the caspase-3 activity. Caspase activity detection is recognised as a standard method for assessing apoptosis^22^. Caspase-3 is activated early during apoptosis. We assayed its activity in polyclonal S2 cells stably expressing *Tn*BVank1 by using the fluorogenic effector caspase substrate Ac-DEVD-ACM. Cell lysates were harvested from S2 cells stably expressing *Tn*BVank1, untransfected S2 cells (negative control), as well as both the positive controls. Caspase-3 activity was significantly increased in S2[ank1] cells compared to the negative control S2 but comparable to positive controls (S2 + CAM and S2[ank1] + CAM) (Fig. 1b). This result suggests that *Tn*BVank1 stimulates the activation of caspase-3 indicating a role of *Tn*BVANK1 in induction of apoptosis in S2 cells.

Moreover, the nuclear morphology, examined by staining with Hoechst 33258, showed intact nuclei appearing light blue in colour in the negative control (S2 cells) while polyclonal S2 cells exhibited bright blue indicating condensed or fragmented nuclei, typical morphological characteristics of apoptosis (see Supplementary Fig. S1).

### Western blot time course

**Figure 2:**
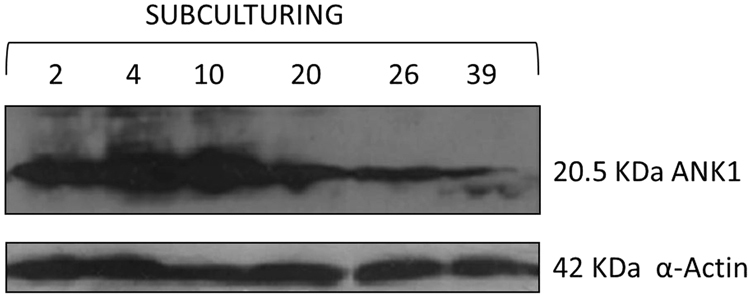
Western blot time course of *Tn*BVANK1 expression. Cell lysates of S2 cells stably expressing *Tn*BVank1 were harvested at subsequent passages (2, 4, 10, 20, 26, 39). Detection of *Tn*BVANK1 was determined by using anti-V5 antibody; each lane was loaded with the same quantity of protein as revealed by the endogenous control, α-Actin. Original Western Blot is reported in Supplementary Information.

To test whether the production of *Tn*BVANK1 decreases over time in the stable polyclonal cell line, as a consequence of apoptotic cell death, a western blot time-course analysis was performed on cell lysates harvested at subsequent splitting passages (2, 4, 10, 20, 26, 39 passages). Western blot analysis showed an effective reduction of *Tn*BVank1 gene expression over time, indicating that the population of cells, stably expressing *Tn*BVank1 within the stable polyclonal cell line, progressively decreases with a concomitant increase of cell number not expressing *Tn*BVank1 (Fig. 2). This result strongly suggest that the expression of the viral gene *Tn*BVank1 induces cell death in S2 cells.

### *In vivo* transient expression of *Tn*BVank1 efficiency in *H*. *virescens* haemocytes

**Figure 3:**
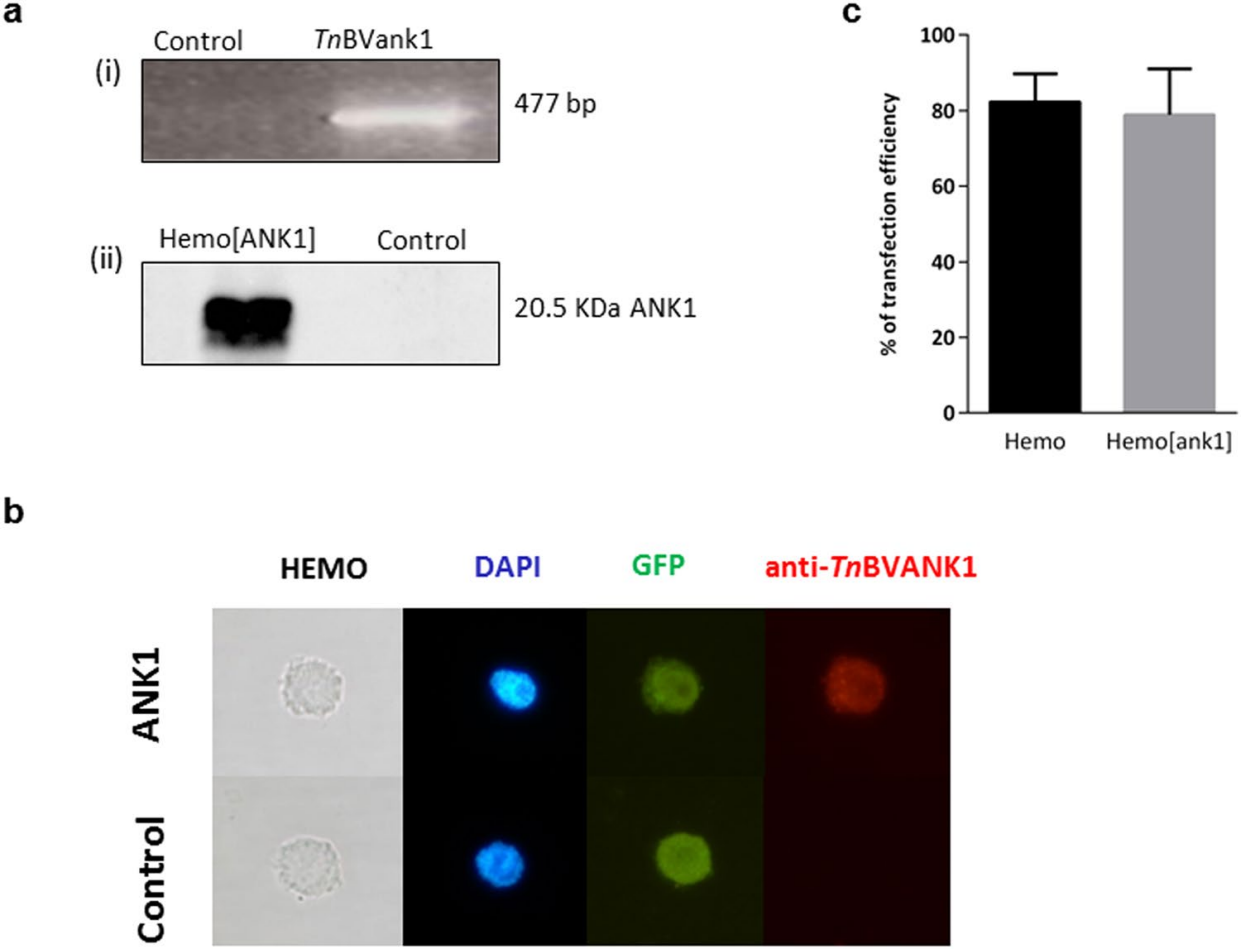
Transient expression of *Tn*BVank1 in *H*. *virescens* haemocytes. (**a**) RT-PCR (i) and western blot (ii) showed expression of *Tn*BVank1 (Hemo[ank1]) and *Tn*BVANK1 protein (Hemo[ANK1]) 56 h after the injection of pIZT/V5-His-ANK1 into haemocytes of early fifth instar *H*. *virescens* larvae. *H*. *virescens* haemocytes extracted from larvae transfected with the empty vector pIZT/V5-His were used as control (Hemo). Original Gel and Western Blot are reported in Supplementary Information. (**b**) Immunofluorescence of *in vivo* transfected haemocytes showed epifluorescent (for GFP) signal in both control (haemocytes transfected with the empty vector) and *Tn*BVANK1 (haemocytes transfected with *Tn*BVank1) (green signal). Red signal of *Tn*BVANK1 was detected for haemocytes transfected with *Tn*BVank1 (ANK1). Nuclei were stained with DAPI (blu signal). Images were observed at immunofluorescence microscopy using Nikon Eclipse 80i equipped with a Nikon Plan Fluor 100x/0.5–1.3 Oil Iris objective, the images were recorded with Nikon Digital Sight DS-U1 camera and ImageJ software. (**c**) Transfection efficiency was measured by counting fluorescent haemocytes 56 h post injection. Each measurement used randomly chosen 100 haemocytes and it was independently replicated three times. Data represent the mean of 3 replicates ± SD. Statistical analysis was performed by one way ANOVA and Tukey’s Test P < 0.05.

Haemocytes exhibited transient expression of *Tn*BVank1 when the recombinant vector pIZT/V5-His-ANK1, mixed with a transfection reagent, was injected into the haemocoel of fifth instar *H*. *virescens* larva (day one). After trying different transfection reagents, and different time after injection, we selected Lipofectin as the most efficient one and 56 h as the best time for *in vivo* transient expression, at least for our experimental conditions. The expression of *Tn*BVank1 transcripts in transfected haemocytes was detected by reverse transcriptase-polymerase chain reaction (RT-PCR) 56 h after injection of pIZT/V5-His-ANK1 using specific primers for *Tn*BVank1 (Fig. 3a-i). The expression of *Tn*BVANK1 protein was assessed by western blot analysis using the anti-V5 antibody (Fig. 3a-ii) and detecting the epifluorescence signal of GFP reporter and the *Tn*BVANK1 protein expression (red signal) by immunofluorescence (Fig. 3b). No signals were detected in RT-PCR, western blot and immunofluorescence experimental controls (haemocytes transfected with the empty pIZT/V5-His vector) except in this last case in which the epifluorescence signal of GFP reporter was detected. Different haemocyte types were successfully transfected and the *in vivo* transfection efficiency was calculated by counting fluorescent haemocytes in the control because of the signal of GFP protein, and in haemocytes *in vivo* transfected (see Supplementary Fig. S2). Approximately 80% of haemocytes expressed the GFP reporter (Hemo) or both the GFP reporter and the viral gene *Tn*BVank1 (Hemo[ank1]), respectively (Fig. 3c).

### *In vivo* transient expression of *Tn*BVank1 and natural parasitism induce apoptosis in *H*. *virescens* haemocytes

**Figure 4:**
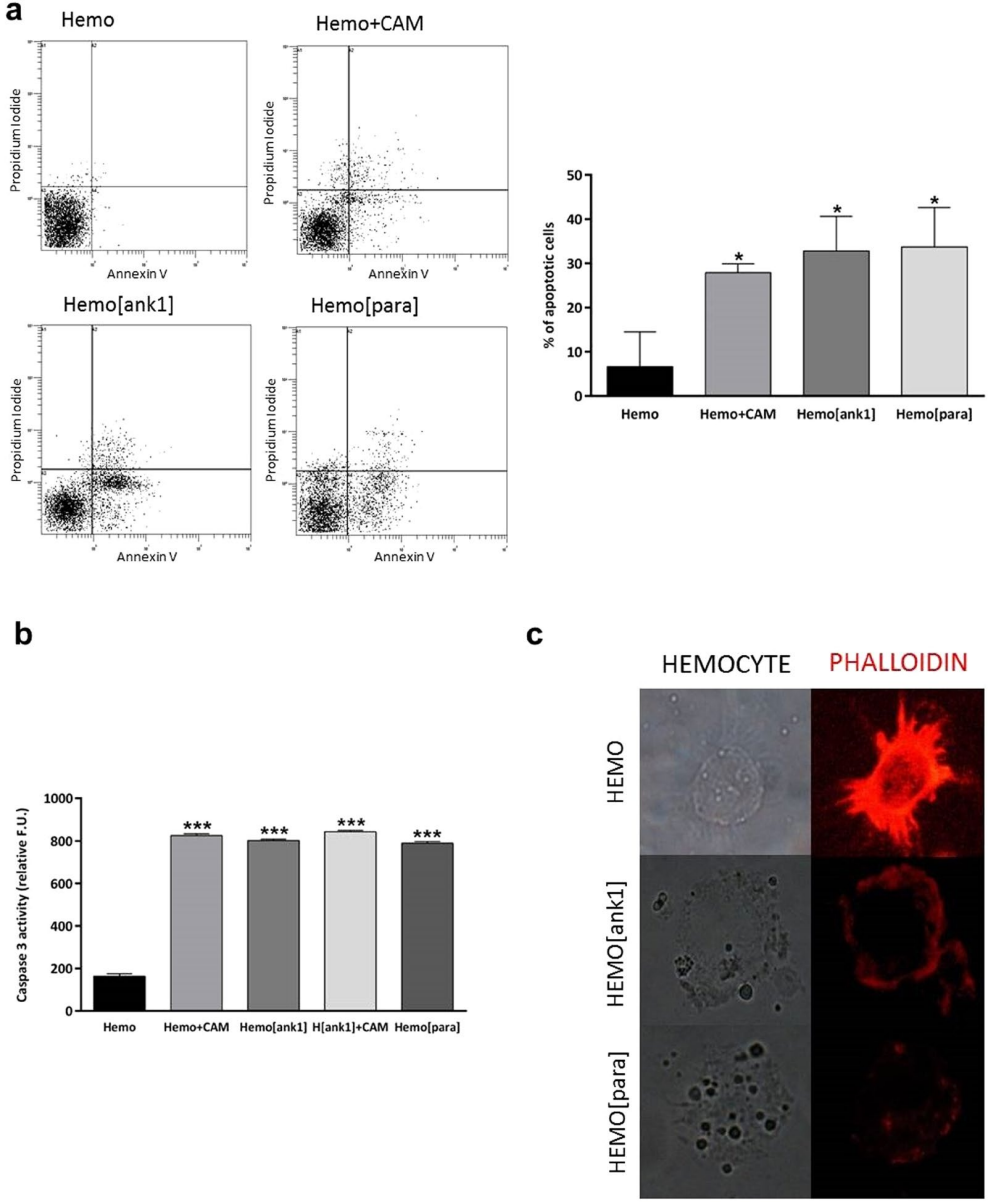
Parasitism and transient expression of *Tn*BVank1 in *H*. *virescens* haemocytes induces apoptosis. (**a**) Flow cytometry analysis on haemocytes collected from parasitised larvae (Hemo[para]) and from *Tn*BVank1 *in vivo* transfected haemocytes (Hemo[ank1]). As negative control haemocytes transfected with the empty pIZT/V5-His vector (Hemo) and as positive control haemocytes treated with camptothecin (Hemo + CAM) were used. Cells were doubly labeled with Annexin V-FITC and PI before analysis by flow cytometry. Dot plots of single positive for Annexin V and double positive for Annexin V and PI cells were interpreted as signs of early and late phases of apoptosis respectively (lower and upper right quadrant respectively). Data are quantified in the bar chart on the right showing the percentage of apoptotic cells. (**b**) Circulating haemocytes were transfected with *Tn*BVank1 gene (Hemo[ank1]) and their caspase-3 activity was compared to the haemocytes (Hemo), transfected with the empty pIZT/V5-His vector (negative control). Apoptosis was induced in positive controls by injecting into haemocoel of the larvae 1 μM camptothecin with the vector pIZT/V5-His with or without *Tn*BVANK1 (Hemo[ank1] + CAM, Hemo + CAM). Caspase-3 activity was determined using Ac-DEVD-AMC as fluorescent substrate. For the assay, 3 × 10^5^ cells were used. Results are expressed as fluorescence (F.U.); an excitation wavelength of 380 nm and an emission wavelength of 430 nm were utilised. Data represent the mean of 3 replicates ± SD. Statistically significant differences between samples are indicated with asterisk (***P ≤ 0.001, one way ANOVA and Tukey’s Test P < 0.05). (**c**) Actin filaments were detected with TRITC-conjugated phalloidin staining in haemocytes collected from parasitised larvae (Hemo[para]), from larvae after *Tn*BVank1 *in vivo* transfection (Hemo[ank1]) and from unparasitised larvae (Hemo). Images were observed at immunofluorescence microscopy using Nikon Eclipse 80i equipped with a Nikon Plan Fluor 100x/0.5–1.3 Oil Iris objective, the images were recorded with Nikon Digital Sight DS-U1 camera and ImageJ software.

We investigated the effect of natural parasitism and of *in vivo* transfected haemocytes expressing *Tn*BVank1 on apoptosis by flow cytometric analysis. Cells undergoing early and late stages of apoptosis were single positive for Annexin V and double positive for Annexin V and PI respectively. Apoptotic cells were detected in haemocytes deriving from parasitised larvae (Hemo[para]) and in haemocytes from *in vivo* transfected larvae (Hemo[ank1]), as well as in the positive control (haemocytes treated with camptothecin). As negative control haemocytes transfected with the empty pIZT/V5-His vector were used and as positive controls, camptothecin (1 μM) was injected into haemocoel of the larvae with the vector pIZT/V5-His either with or without *Tn*BVank1 (Fig. 4a).

To further investigate apoptosis with same haemocytes samples, the caspase-3 activity was measured, using the fluorogenic effector caspase substrate Ac-DEVD-ACM. Haemocytes transfected with *Tn*BVank1 (Hemo[ank1]) and haemocytes deriving from parasitised larvae (Hemo[para]) showed a strong increase of caspase-3 activity compared to haemocytes transfected with the empty pIZT/V5-His vector (negative control) (Hemo) (Fig. 4b).

Moreover, phalloidin staining showed actin filaments grouped in bundles under the membrane both in haemocytes from parasitised larvae and in *Tn*BVank1 transfected haemocytes (Fig. 4c). The percentage of apoptotic cells, the caspase-3 activity and the phalloidin staining were comparable in parasitised haemocytes and in *Tn*BVank1 transfected haemocytes. These results suggest that the apoptosis does not seem to be due to an overexpression of the gene *Tn*BVank1 after *in vivo* transfection respect to its expression in haemocytes after parasitisation. Indeed, when a qPCR on haemocytes collected after *in vivo* transfection with *Tn*BVank1 and after parasitisation was performed, results did not show significant differences of *Tn*BVank1 transcript expression level after parasitisation and after *in vivo* transfection (see Supplementary Fig. S3).

Also the nuclei staining with Hoechst 33342 revealed the occurring of apoptosis in *H*. *virescens* haemocytes transfected with *Tn*BVank1 (Hemo[ank1]). The cells in the haemocytes transfected with the empty pIZT/V5-His vector (negative control) (Hemo) showed roundshaped and homogeneously stained nuclei, whereas haemocytes transfected with *Tn*BVank1 (Hemo[ank1]) showed accumulation of fluorescent dye indicating obvious chromatin condensation and fragmentation (see Supplementary Fig. S1).

### Functional proteomics experiments

Isolation of *Tn*BVANK1 complexes was performed on a total protein extract from S2 cells stably expressing a V5-tagged form of the protein. Following pre-cleaning treatment, the unbound fraction was immunoprecipitated with an anti-V5 specific antibody in order to capture *Tn*BVANK1 functional complexes. The occurrence of *Tn*BVANK1 protein within the immunoprecipitated material was detected by western blot using anti-V5 antibody (see Supplementary Fig. S4a). Immunoprecipitated complexes were fractionated by SDS-PAGE (see Supplementary Fig. S4b) and protein bands, excised from the gel, were identified by LC-MS/MS analysis. Protein bands from the control S2 cells were also analysed as control. Putative *Tn*BVANK1 protein interactors were obtained by differential analysis comparing the proteins identified in S2 cells stably expressing *Tn*BVank1 and those from the control S2 cells.

The results of this functional proteomic approach are summarised in Supplementary Table S1. As the experiment was performed on the *Drosophila* S2 cell line, putative interactors were annotated against the *Drosophila melanogaster* genome and then compared to *Homo sapiens* proteins. Among interactor proteins, NF-Κb p110 subunit was identified, confirming previous studies showing interaction between *Tn*BVANK1 and the transcription factor NF-κB^8^.

Moreover, we found several proteins involved in the apoptotic pathway, as the transcription factor Stwl^23^, the mitochondrial carrier homolog 1 (Mtch)^24^, ALG-2 interacting protein X^21^ and the GTPase Rab1^25,26^. Among these proteins, we focused our attention on the ALG-2 interacting protein X, also called Alix. This protein was chosen on the basis of its involvement in programmed cell death, for subsequent analyses.

### *Tn*BVANK1 protein colocalises with Alix protein in polyclonal S2 cell line

**Figure 5:**
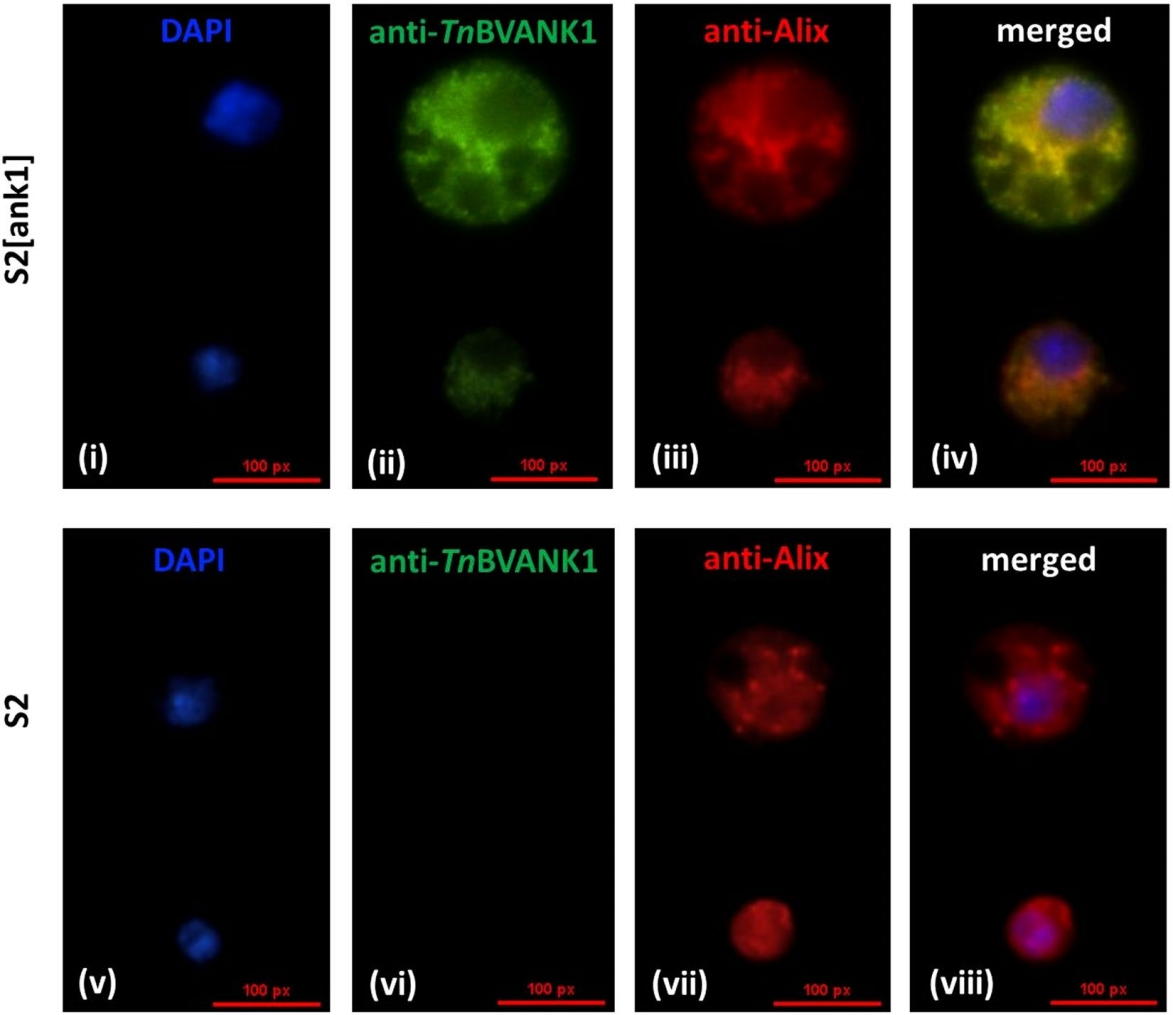
*Tn*BVANK1 protein colocalises with Alix in polyclonal S2 cell line. i-iv) Polyclonal S2 cell line coexpressing both *Tn*BVANK1 and Alix proteins stained with anti-*Tn*BVANK1 and anti-Alix antibodies. v-viii**)** Staining of control S2 cells expressing only Alix protein. The localisation of *Tn*BVANK1 and Alix proteins was determined by FITC (green signal) and TRITC (red signal) fluorescence, respectively and nucleus was stained with DAPI (blue signal). In merged images there is a clear overlap of red and green signals showing a colocalisation (yellow signal) of analysed proteins. Images were observed at immunofluorescence microscopy using Nikon Eclipse 80i equipped with a Nikon Plan Fluor 100x/0.5–1.3 Oil Iris objective, the images were recorded with Nikon Digital Sight DS-U1 camera and ImageJ software.

To further support the interaction between *Tn*BVANK1 and Alix, the localization of both these proteins was examined in polyclonal S2 cell line by immunofluorescence labeling using both anti-Alix and anti-*Tn*BVANK1 antibodies. Figure 5(ii,iii) shows that both *Tn*BVANK1 (green signal) and Alix (red signal) are widely distributed throughout the cytoplasm of polyclonal S2 cell line and the merged picture (Fig. 5-iv) indicates that the two proteins seem to colocalise, as there is a clear overlap of red and green signals of analysed proteins. This result further supports the potential to interact of the two proteins as demonstrated by functional proteomics experiments. In the control, represented by *Drosophila* S2 cells, as expected, no signal of *Tn*BVANK1 was detected while Alix signal is present in the cytoplasm as in polyclonal S2 cells. This finding provides a further element supporting the hypothesis of the interaction between these two proteins.

### *In vitro* and *in vivo* silencing of Alix suppresses caspase-3 activity induced by *Tn*BVank1

In order to assess whether the apoptosis induced by *Tn*BVank1 is mediated by the interaction with Alix, we silenced the Alix gene transcript by RNAi both in the S2 cell line stably expressing *Tn*BVank1 and in *H*. *virescens* circulating haemocytes transiently expressing *Tn*BVank1.

**Figure 6:**
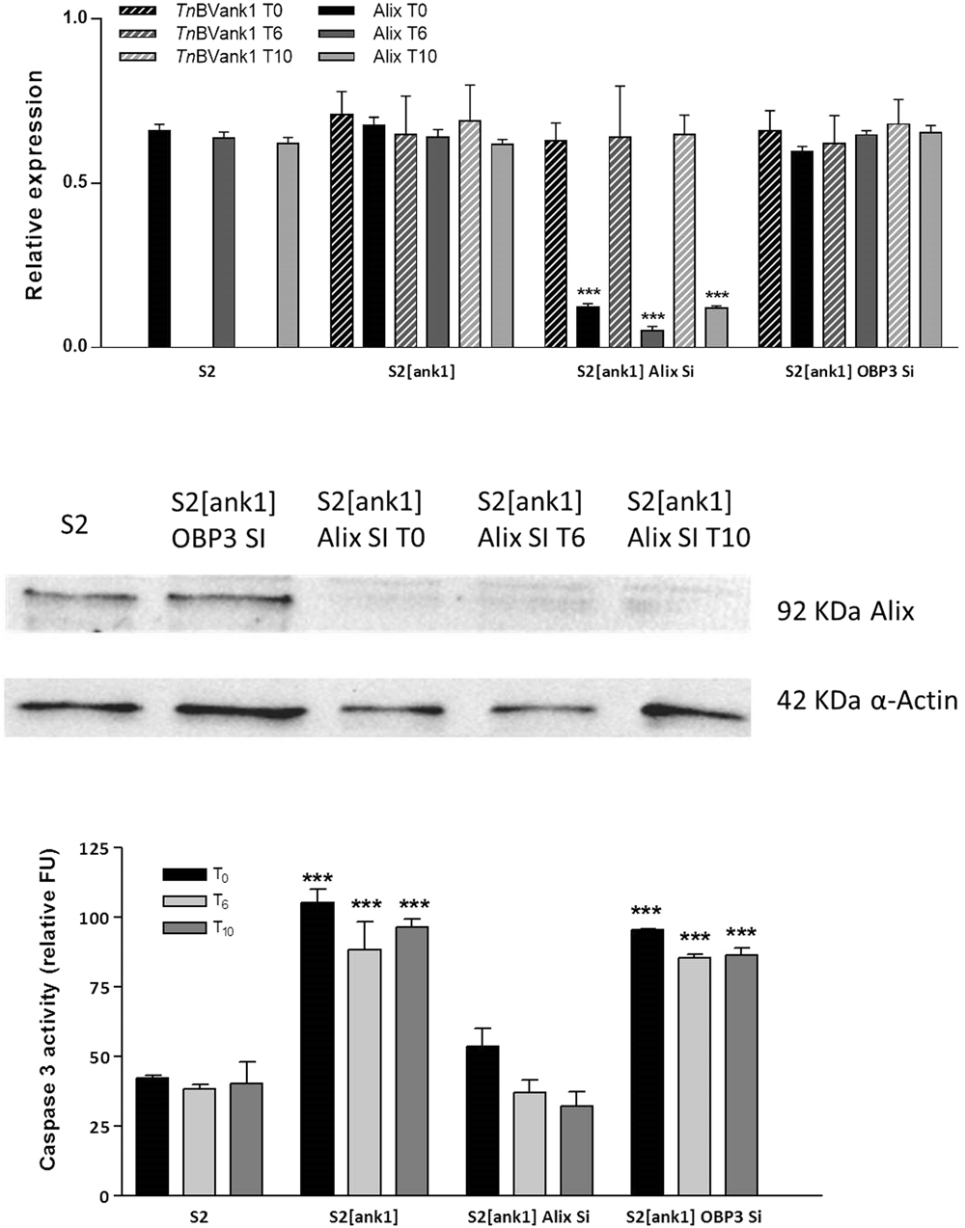
Alix and *Tn*BVank1 transcript levels in S2 cells and post-Alix silencing caspase-3 assay. (**a**) Expression levels of Alix and *Tn*BVank1 at different hours (T_0_, T_6_, T_10_) after Alix silencing in S2 cells stably expressing *Tn*BVank1 (S2[ank1] Alix Si), normalised to the endogenous control Rpl32^54^. As control ds*Apis*OBP3 was used (S2[ank1] OBP3 Si). No expression of *Tn*BVank1 was observed in the control S2 cells. (**b**) Western blot of Alix expression at different hours (T_0_, T_6_, T_10_) after Alix silencing in S2 cells stably expressing *Tn*BVank1. Original Western Blot is reported in Supplementary Information. (**c**) Post-Alix silencing caspase-3 activity in S2 cells, S2 cells stably expressing *Tn*BVank1 (S2[ank1]), S2 cells stably expressing *Tn*BVank1 with silenced Alix transcript (S2[ank1] Alix Si) and S2 cells stably expressing *Tn*BVank1 transfected with dsRNA of *Apis*OBP3 as control (S2[ank1] OBP3 Si), harvested at different times (0, 6 and 10 h) after the replacement of the medium containing dsRNA. Data represent the mean of 3 replicates ± SD. Statistically significant differences between samples are indicated with asterisk (***P ≤ 0.001, one way ANOVA and Tukey’s Test P < 0.05).

**Figure 7:**
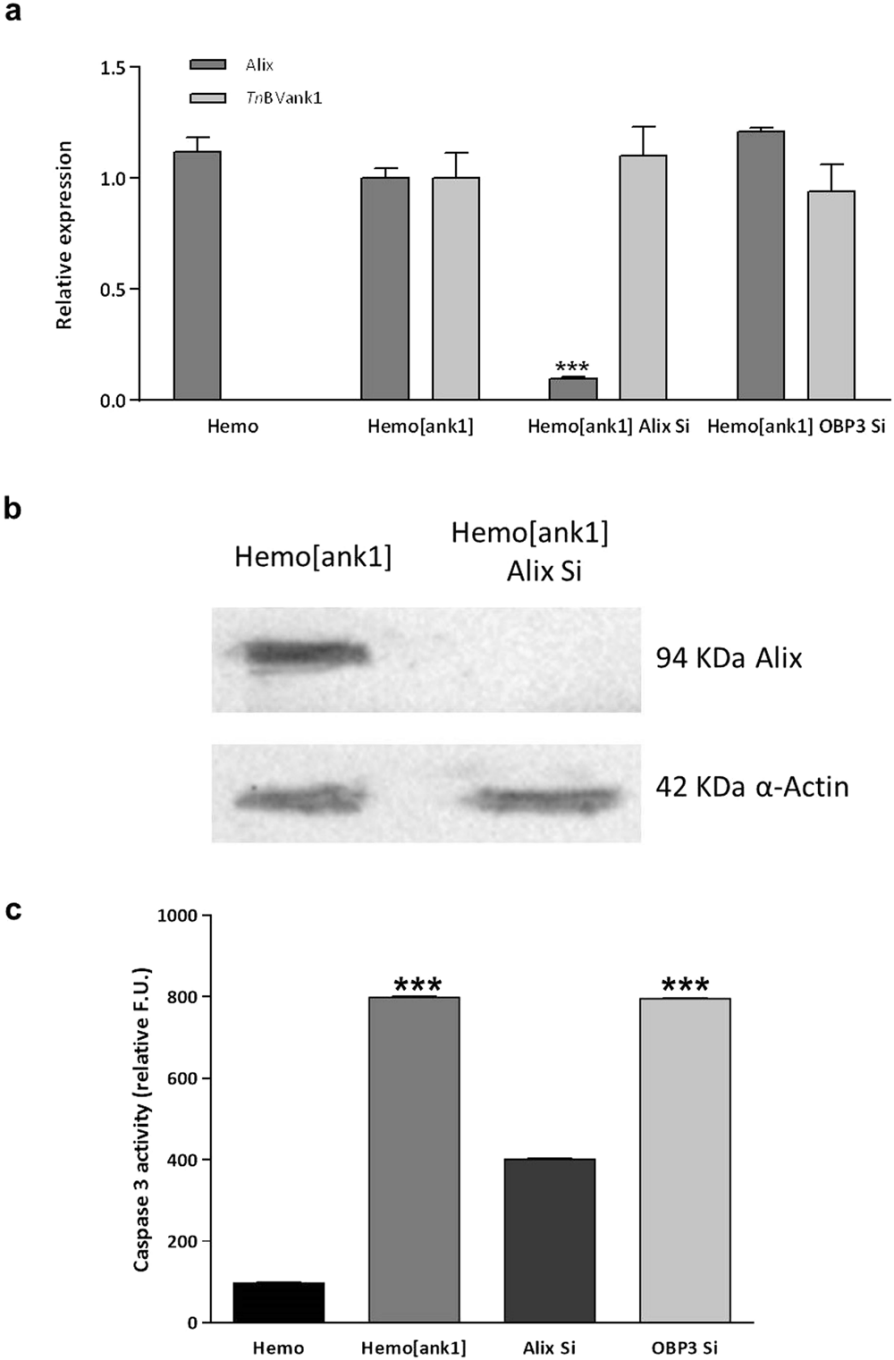
Alix and *Tn*BVank1 transcript levels in *H*. *virescens* haemocytes and post-Alix silencing caspase-3 assay. (**a**) Expression level of Alix and *Tn*BVank1 transcripts into *H*. *virescens* haemocytes 56 h after Alix silencing (Hemo[ank1] Alix Si), normalised to the endogenous controls (EF1α and RP13^49^). No expression of *Tn*BVank1 was observed in the control haemocytes transfected with the empty pIZT/V5-His vector (Hemo). (**b**) Western blot of Alix expression in haemocytes 56 h after Alix silencing and transfected with *Tn*BVank1(Hemo[ank1] Alix Si); as control haemocytes were transfected with the empty vector (Hemo). Original Western Blot is reported in Supplementary Information. (**c**) Post-Alix silencing caspase-3 activity in haemocytes, transfected with *Tn*BVank1 gene (Hemo[ank1]), cotransfected with Alix dsRNA and *Tn*BVank1 (Hemo[ank1] Alix Si). As control haemocytes were transfected with the empty vector (Hemo) and cotransfected with *Apis*OBP3 dsRNA and *Tn*BVank1 (Hemo[ank1] OBP3 Si). Data represent the mean of 3 replicates ± SD. Statistically significant differences between samples are indicated with asterisk (***P ≤ 0.001, one way ANOVA and Tukey’s Test P < 0.05).

The success of RNAi was verified by quantitative Real time PCR (qPCR) and western blot performed on polyclonal S2 cells stably expressing *Tn*BVank1, harvested at different time points (0, 6, and 10 h) (Fig. 6a and b) and on *H*. *virescens* haemocytes transiently expressing *Tn*BVank1 (Fig. 7a and b) both treated with Alix dsRNA. The cells in which Alix was silenced (S2[ank1] Alix Si and Hemo[ank1] Alix Si) showed that the available transcript of Alix gene was significantly reduced compared to its expression in S2 cell line, in S2 cell line stably expressing *Tn*BVank1 (S2[ank1]) and in S2 cell line stably expressing *Tn*BVank1 treated with the ds*Apis*OBP3 (S2[ank1] OBP3 Si) as well as in circulating haemocytes transiently transfected with the empty pIZT/V5-His vector (Hemo), in haemocytes transiently expressing *Tn*BVank1 (Hemo[ank1]) and in haemocytes transiently expressing *Tn*BVank1 treated with the ds*Apis*OBP3 (Hemo[ank1] OBP3 Si) (Fig. 7a).

These results were confirmed by western blot analysis showing that Alix protein expression was reduced in S2 cells and haemocytes after RNAi experiments (Figs 6b and 7b).

Same samples were tested by qPCR for their expression of *Tn*BVank1 transcript demonstrating its expression in all described conditions except in the case of S2 cell line and in circulating haemocytes transiently transfected with the empty pIZT/V5-His vector (Figs 6a and 7b).

Caspase-3 assay performed on same samples analysed by qPCR as listed above indicated that in S2 cell line stably expressing *Tn*BVank1 and silenced for Alix transcript, caspase-3 activity was highly reduced compared to the S2 cell line stably expressing *Tn*BVank1 (Fig. 6c). Similar results were obtained *in vivo*, since caspase-3 activity measured in *H*. *virescens* haemocytes cotransfected with Alix dsRNA and *Tn*BVank1 was significantly reduced compared to haemocytes transfected with *Tn*BVank1 alone (Fig. 7c). Taken together, the *in vivo* and *in vitro* experiments suggest that Alix/*Tn*BVANK1 interaction strongly contributes in inducing apoptosis both in S2 cell line and in circulating haemocytes.

## Discussion

The bracovirus associated with the wasp *Toxoneuron nigriceps* (*Tn*BV) is one of parasitic regulatory factors of maternal origin involved in immunosuppression and alteration of endocrine balance of the host *Heliothis virescens* ^1,4,12^. A large number of studies have been addressed to the elucidation of parasitised host developmental syndromes^6,7,18,27^. Briefly, in this experimental model, parasitised last instar larvae fail to pupate, allowing a suitable nutritional substrate for parasitoid larvae over an extended time period^28^. Here, we report experimental evidence demonstrating the role of *Tn*BVank1, a viral gene member of the *Tn*BV ankyrin (ank) gene family^8^, in inducing apoptosis processes in a stably *Tn*BVank1 transfected polyclonal insect cell line (*Drosophila* Schneider’s S2 cells) and in circulating haemocytes of *H*. *virescens* larvae transiently transfected with *Tn*BVank1 gene, thus reproducing the apoptotic effects observed in haemocytes of parasitised *H*. *virescens* larvae. *Tn*BVank1, together with *Tn*BVank2 and *Tn*BVank3, belongs to the *Tn*BVank family^8^ and displays significant sequence similarity with members of the IκB family, that are proteins generally involved in the control of NF-κB signaling pathways both in insects and in vertebrates^29^.

IκB members are characterised by repeated sequences of ankyrins that bind to transcription factors NF-κB/Rel, masking their nuclear localization signal. Upstream sequences of ankyrin serine residues can be phosphorylated by the IκB kinase (IκK) in response to a number of extracellular signals^29^. Once phosphorylated, IκB can be released and degraded via the proteasome, allowing translocation of the transcription factor NF-κB/Rel in the nucleus and subsequent activation of target genes^29^. In *Drosophila*, the IκB-like protein regulates various cellular responses triggered by nuclear transfer protein NF-κB/Rel, such as the embryonic development and the release of defense-related antimicrobial peptides^29^.

The gene family of viral ankyrins (also called *vankyrin*), is widespread among both Bracovirus and Ichnovirus, and each genome has several copies of these genes^8,15,30–32^. All known polydnavirus (PDV) ankyrin genes lack the regulatory sites, serine residues at the N-terminus and PEST domain at the C-terminal end^13,15,33–36^. These peculiar features strongly suggest that viral ankyrins may act as inhibitors by irreversibly binding and retaining NF-κB in the cytoplasm thus affecting a variety of cellular responses in parasitised hosts, including alteration of innate immune defenses^8,14,15^. This mechanism, proposed to explain the alteration of the host immune response induced by the parasitoid, is very similar to the strategy used by ASFV (African swine fever virus) to inhibit the activation of NF-κB transcription factors in mammals. The ASFV IκB-like protein irreversibly binds NF-κB, preventing its entry into the nucleus^37^, similarly to the effect observed for *Tn*BVank1^8^.

The genome of *Campoletis sonorensis* Ichnovirus (*Cs*IV) contains seven copies of ankyrin genes named vankyrin^33^. Among these vankyrins, P-vank-1 is an interesting viral factor, as it, when is stably expressed in eukaryotic Sf9 insect cells, seems to have a role in protecting cells from apoptosis^34^. The role of vankyrin P-vank-1, which belongs to the same family as *Tn*BVank1, led us to hypothesise a possible involvement of *Tn*BVank1 in the regulation of programmed cell death. Here we have shown that, unlike vankyrin P-vank1, the viral gene *Tn*BVank1 induces apoptosis. The different biological roles of these proteins are surprising but not unusual; it is reported that the vankyrin I^2^-vank-3 of *Campoletis sonorensis* Ichnovirus (*Cs*IV), although highly similar to P-vank-1 (with 83% amino acid sequence identity), plays a different role^36^. This could be also explained by their differential expression and localisation in the host tissues.

In the polyclonal S2 cell line stably expressing *Tn*BVank1, a significant increase of apoptotic cell number, compared to untreated S2 cells (negative control), was observed by flow cytometry when doubly stained with Annexin V–FITC and PI. Moreover, compared to negative control, in the polyclonal S2 cell line stably expressing *Tn*BVank1 a significantly higher caspase-3 activity and apoptotic nuclei were detected. A previous work^16^ revealed typical morphological modifications of apoptosis in parasitised *H*. *virescens* granulocytes (a subpopulation of haemocytes). Here we confirmed that haemocytes undergo apoptosis after natural parasitism, by flow cytometry analysis, caspase-3 assay and phalloidin staining. This result led us to deepen the role of *Tn*BVank1 in apoptotic pathway in haemocytes of non parasitised larvae by *in vivo* transient transfection that is an useful method to determine the biological function of a foreign gene. We observed an apoptotic profile by flow cytometry, a significantly increased caspase-3 activity and nuclear morphological changes in *H*. *virescens* haemocytes after *Tn*BVank1 *in vivo* transfection. These results demonstrate that *Tn*BVank1 is one of the Polydnavirus genes responsible for apoptosis observed in *H*. *virescens* haemocytes after parasitisation^16^. A qPCR performed on both haemocytes derived from parasitised host and *Tn*BVank1 *in vivo* transient transfected haemocytes evidenced that the expression level of *Tn*BVank1 after transient expression was comparable to its expression after natural parasitism, thus demonstrating that the observed apoptotic effect is not due to the result of an unnatural overexpression of the protein.

*Tn*BVank1 would be the first viral ankyrin that, besides preventing the nuclear translocation of NF-κB^8^ thus disrupting cell pathways under its control^15,29,38^, is also able to directly induce apoptosis. *Tn*BVank1 is not the first *Tn*BV gene that induces apoptosis. Lapointe and colleagues^18^ showed that the gene *Tn*BV1 induces apoptosis when expressed in cell lines derived from *Spodoptera frugiperda* (SF21) and *Tricoplusia ni* (High Five). However, when the authors tried the *in vivo* expression of *Tn*BV1-recombinant baculovirus, no apoptotic effect was detected, leaving the role of *Tn*BV1 expression in parasitised *H*. *virescens* larvae elusive. *Tn*BV1 protein may interact with other *Tn*BV proteins during parasitism to exert its function in the apoptotic pathway^18^. In contrast, our results indicate that a single viral gene, *Tn*BVank1, is able to induce apoptosis, *in vitro* and *in vivo*, without the requirement of other virus-encoded genes as interaction partners. The induction of apoptosis is one of the most common strategies adopted by various PDVs to suppress the immune response^39^. However, the parasitism do not affect all the cells of the immune system^16^ probably as a strategy to allow the host to respond to a possible further pathogen invasions ensuring the development of wasp embryo^39,40^.

The molecular mechanisms of apoptosis induction exerted by *Tn*BVank1 were more closely investigated by functional proteomic approaches. Multiprotein complexes comprising *Tn*BVANK1 were isolated from polyclonal S2 stably expressing *Tn*BVank1 by immunoprecipitation and the individual protein components were identified by mass spectrometry. Among the various *Tn*BVANK1 protein partners, we focused our attention on Alix (ALG-2 interacting protein X) as this multifunctional protein is involved in several protein interaction networks, including programmed cell death, endocytic membrane trafficking and cytoskeletal remodeling^41,42^. To confirm the possible interaction between *Tn*BVANK1 and Alix proteins, polyclonal S2 cell line was examined by coimmunolocalisation experiments, using anti-*Tn*BVANK1 and anti-Alix antibodies. Observations by immunofluorescence microscopy revealed that *Tn*BVANK1 are both present in the cytoplasm and seems to colocalises with Alix protein.

The role of *Tn*BVANK1-Alix interaction in apoptosis was further investigated by silencing experiments both *in vitro* and *in vivo*. Silencing of Alix expression in *Tn*BVank1 stably transfected S2 cells resulted in a significant reduction of apoptosis assessed by caspase-3 activity. Similarly, silencing Alix in circulating haemocytes of *H*. *virescens* larvae transiently transfected with *Tn*BVank1 drastically reduced caspase-3 activity. In both cases, the *Tn*BVank1 transcript expression level evaluated by qPCR remained constant.

These *in vitro* and *in vivo* results suggest that *Tn*BVank1 induces apoptosis through interaction with Alix.

The interaction between *Tn*BVANK1 and Alix was also reported by a recent study showing their colocalisation in prothoracic glands of a *Drosophila melanogaster* mutated strain expressing *Tn*BVank1. It is reported that *Tn*BVANK1 impairs the ecdysone biosynthesis and causes the developmental arrest by altering the vesicular traffic of ecdysteroid precursors in the prothoracic gland steroidogenic cells of *Drosophila melanogaster*. The authors showed that the *Tn*BVank1 affects the sterol delivery interacting with Alix that is also involved in controlling the cholesterol export from endosomes^16,43^.

Although the molecular mechanisms are unclear, Alix cooperating with ALG-2 is involved in the induction of apoptosis through both intrinsic and extrinsic apoptotic pathways^41^. A possible way of induction of apoptosis by the protein Alix is via TNF-R1 (TNF receptor-1), which contains a DD (Death Domain) capable of activating caspase 8, an initiator of apoptotic process. The interaction TNF-R1/Caspase 8 initiates the apoptotic cascade. This mechanism requires that Alix forms a complex with TNF-R1^44^.

Our data show that *Tn*BVANK1 strongly induces apoptosis interacting with Alix, via the mitochondrial apoptotic pathway. Moreover, the previously demonstrated role of *Tn*BVank1 in the control of the NF-κB signaling^8^ preventing its nuclear translocation, could also further increase the sensitivity of the cell to the cell death signals^38^.

This seems like an effective strategy of the virus to disable the host immune system and facilitate successful development of its associated wasp.

## Materials and Methods

### Insect cells culture

*Drosophila* Schneider’s S2 cells (Life Technologies, Carlsbad, CA, USA) were cultured in Schneider’s *Drosophila* medium (WVR, Radnor, PA, USA) supplemented with 10% heat inactivated fetal bovine serum (Life Technologies, Carlsbad, CA, USA) and were maintained in 75 cm^2^ tissue culture flasks (Corning, NY, USA) at 27 °C.

### Stable transfection of *Tn*BVank1 in S2 cells

The *Tn*BVank1 cDNA, obtained as previously described by Falabella and colleagues^8^, was amplified with specific primers containing XbaI and SacII enzyme restriction sites (underlined):

Ank1F 5′-CTAGTCTAGAATGGAAAACTCATTACTCATTG-3′

Ank1R 5′-TCCCCGCGGATTATCATCACACTTAGCGCC-3′

The obtained amplimer was cloned into TOPO vector (Life Technologies, Carlsbad, CA, USA) and sequenced. The insert was then released by XbaI and SacII restriction enzyme digestion (underlined) and cloned into the expression vector pIZT/V5-His (Life Technologies, Carlsbad, CA, USA). This vector uses the OpIE2 promoter from the *Orgyia pseudotsugata* baculovirus for constitutive expression of the gene of interest and encodes a zeocin-green fluorescent protein (GFP) gene fusion under control of the OpIE1 promoter. The 3′ ends of the Ank1 fragments were cloned in frame with a “V5 epitope” and with a tail of six histidines. The obtained construct was sequenced, verified and transiently expressed in S2 cells by cationic lipid-mediated transfection. 24 h prior transfection S2 cells were seeded at 70 to 80% confluency (0.5 × 10^6^) in 12-well culture plates (Corning, NY, USA) and subsequently transfected using 8 μl of Cellfectin (Life Technologies, Carlsbad, CA, USA) and 1 μg of DNA per 1 ml of medium and incubated at 27 °C for 6 h. After incubation, the medium was removed and cells were re-incubated with 1 ml of Schneider’s *Drosophila* complete medium containing 10% FBS at 27 °C for 48 h. Stably transformed cells were selected by adding 400 μg/ml of zeocin (Life Technologies, Carlsbad, CA, USA). Populations of antibiotic-resistant cells were amplified to obtain polyclonal S2 cell lines stably expressing *Tn*BVank1.

### Insect rearing

*Heliothis virescens* larvae were maintained on an artificial diet as previously described^28^. *Toxoneuron nigriceps* was reared in the laboratory, on larval stages of its host, *H*. *virescens*, according to Vinson *et al*.^45^. Rearing temperature was 29 ± 1 °C for the host, parasitised host larvae and cocoons. *T*. *nigriceps* adults were kept at 25 ± 1 °C and fed with water and honey. A 16 h light photoperiod was adopted and the relative humidity was 70 ± 5%. *H*. *virescens* last instar larvae were staged according to Webb & Dahlman^46^ and synchronised as reported by Pennacchio *et al*.^47^.

For experimental procedures, described below, *H*. *virescens* larvae were parasitised at the day one of the last larval instar.

### *In vivo* transient expression of *Tn*BVank1 in *H*. *virescens* haemocytes

For *in vivo* transfection of pIZT/V5-His-ANK1, Lipofectin reagent (Life Technologies, Carlsbad, CA, USA) was used. pIZT/V5-His-ANK1 (0.5 µg) was mixed with 4 µl of transfection reagent and incubated for 20 min at room temperature for the formation of DNA-lipid complex before injection into the haemocoel of *H*. *virescens* fifth instar larvae (day one) using a Hamilton syringe (Sigma-Aldrich St Louis, MO, USA). Before injection, larvae were anaesthetised by immersion in sterile ice water. Haemocytes were collected as described by Ferrarese and colleagues^16^ 56 h post-transfection to perform all the biological assays. This time was selected as the most successful for *Tn*BVANK1 expression, tested after a time course at 24 h, 48 h and 56 h post injection (data not shown).

Success of the haemocytes transfection was analysed by reverse transcriptase-polymerase chain reaction, and the protein expression was detected by western blot analysis using anti-V5 antibody (Life Technologies, Carlsbad, CA, USA) and by immunofluorescence experiments. To assess whether *Tn*BVANK1 induces apoptosis *in vivo*, the caspase-3 assay was performed on haemocytes as describe below.

### Reverse transcriptase-polymerase chain reaction

Total RNA was extracted from *H*. *virescens* haemocytes, previously transfected with the vector pIZT/V5-His (as negative control) or recombinant vector expressing *Tn*BVank1, by using TRI Reagent (Sigma-Aldrich St Louis, MO, USA). Concentration and purity of the total RNA samples were measured using the NanoDrop ND-1000 Spectrophotometer (ND-1000, Thermo Scientific, Waltham, MA, USA). To remove DNA contamination, the samples were treated with 1U of DNase I (Life Technologies, Carlsbad, CA, USA) per microgram of RNA, for 15 min at room temperature. The reaction was stopped by adding 1 µl of 25 mmol/L EDTA and incubating at 65 °C for 10 min. The complementary DNA was synthesised by SuperScript III Reverse Transcriptase (Life Technologies, Carlsbad, CA, USA) according to the manufacturer’s protocol. The cDNA was used as a template for PCR amplification using the following primers:

Ank1F 5′-ATGGAAAACTCATTACTCATTG-3′

Ank1R 5′-ATTATCATCACACTTAGCGCC-3′

To confirm no vector DNA contamination was present after RNA extraction, an aliquot of the isolated RNA was directly used as a template for a test PCR (i.e. without reverse transcription), which showed no PCR product.

### *In vivo* transient transfection efficiency

*Heliothis virescens* haemocytes were isolated 56 h after injection of the vector pIZT/V5-His with or without *Tn*BVank1 as described above. Haemocytes were fixed for 10 min at room temperature in 4% formaldehyde in 1X phosphate buffer saline (PBS) pH 7.5 and then permeabilised for 30 min with PBS containing 0.1% Triton X-100. The cells were blocked with 1% bovine serum albumin (BSA) for 30 min and, subsequently, only the haemocytes transfected with *Tn*BVank1 were incubated with the primary antibody, anti-*Tn*BVANK1^48^ diluted 1:200 in BSA 1%, in a humidified chamber at 4 °C overnight. After three washes in PBS 1X, the cells were incubated with the secondary antibody TRITC-conjugated (Sigma-Aldrich St Louis, MO, USA), 1:1000 in 1% BSA for 1 h at room temperature in dark condition. After washing in PBS 1X three times, the slides were mounted with Fluoroshield^TM^ with DAPI, histology mounting medium (Sigma-Aldrich St Louis, MO, USA).

To measure the total cell fluorescence, the slides were observed microscopically with Nikon Eclipse 80i equipped with a Nikon Plan Fluor 100x/0.5–1.3 Oil Iris objective and the images were recorded with Nikon Digital Sight DS-U1 camera. The intensity of the cell fluorescence was measured using the ImageJ software (http://rsbweb.nih.gov/i j/).

The GFP protein expression was analysed directly by counting fluorescent haemocytes compared to total haemocytes. A hundred haemocytes from a randomly selected field of view at 40x magnification were analysed for each measurement. Each treatment was independently replicated three times.

### *Tn*BVank1 expression in haemocytes after *in vivo* transfection and after parasitism

To evaluate *Tn*BVank1 expression in haemocytes both *in vivo* transfected and after parasitism, quantitative RT-PCR (qPCR) experiments were carried out. RNA extraction and cDNA synthesis were conducted as previously described. PCR amplification was performed using GoTaq qPCR Master Mix (Promega, Fitchburg, WI, USA) on an ABI PRISM® 7500 Fast Real-Time PCR System Thermal Cycler (Applied Biosystems, USA) and in a 20 μl final volume. Sequence specific primers, each in a concentration of 0.3 μM, were:

Ank1F 5′-ATCATGAGGGGCATGCATG-3′

Ank1R 5′-AGCACCAGCGCTGTGAAGT-3′

As reference genes we used the *H*. *virescens* genes coding for the Elongation Factor 1-alpha (EF1α) and 60S Ribosomal Protein 13 (RP13)^49^ (see Supplementary Fig S5). Primers were:

EF1αF 5′-TTGAAGCCTGGTACCATCGT-3′

EF1αR 5′-GTTGGGTGGGTTGTTCTTGG-3′

RP13F 5′-TCGTGGTAAGGTGAAGGCAT-3′

RP13R 5′-AGTCACAGCCTCAACGATCT-3′

All qPCR analyses were performed for a set of 3 biological replicates. Quantification analysis of amplification was done using the comparative C_T_ (ΔC_T_) method. The efficiencies of the three amplicons were approximately equal (Ank1 = 0.892159; EF1α = 0.914590; Rp13 = 0.896174).

### *In vitro* and *in vivo* flow cytometric analysis

Apoptosis was evaluated by flow cytometric analysis of Annexin V and Propidium Iodide (PI)-stained cells using fluorescein isothiocyanate (FITC) Annexin V Apoptosis Detection kit I (Becton Dickinson, Biosciences, San Jose, CA, USA). The assay was carried out on S2 cells (negative control), on S2 cells stably expressing *Tn*BVank1 and on *H*. *virescens* haemocytes after *Tn*BVank1 *in vivo* transfection and after parasitism. S2 cells and polyclonal S2 cells were used in passages 4–10. For the positive control, apoptosis was induced by treating the cells with 1 μM camptothecin, a cytotoxic alkaloid inhibitor of Topoisomerase I, known to induce apoptosis^50,51^ (Sigma-Aldrich St Louis, MO, USA), or injecting into haemocoel of larvae directly 1 μM camptothecin for 20 h^34^. As negative control for the *in vivo* assay, *H*. *virescens* haemocytes after injection of the vector pIZT/V5-His without *Tn*BVank1 were used. Cell samples were washed twice and incubated with Annexin V-FITC/PI for 15 min as manufacturer’s protocol. Stained samples were acquired using Navios flow cytometer and analysed by Kaluza Analysis 1.3 software (Beckman Coulter). Single positive for Annexin V and double positive for Annexin V and PI cells were interpreted as signs of early and late phases of apoptosis respectively.

### *In vitro* and *in vivo* Caspase-3 activity assay

The caspase-3 fluorimetric assay was carried out on S2 cells (negative control), on S2 cells stably expressing *Tn*BVank1 and on *H*. *virescens* haemocytes after *Tn*BVank1 *in vivo* transfection. S2 cells and polycnonal S2 cells were used in passages 4–10. For the positive control, apoptosis was induced by treating the cells with 1 μM camptothecin^50,51^ (Sigma-Aldrich St Louis, MO, USA) or injecting into haemocoel of larvae directly 1 μM camptothecin for 20 h^34^. As negative control for the *in vivo* assay, *H*. *virescens* haemocytes after injection of the vector pIZT/V5-His without *Tn*BVank1 were used. As a substrate of caspase-3 the Ac-DEVD-AMC caspase-3 fluorogenic substrate (BD Biosciences Pharmingen, San Diego, CA, USA) was used. The assay protocol requires primarily lysis of cells. Cells were centrifuged at 1000 *g* for 10 min and then resuspended with Cell Lysis Buffer (10 mM Tris-HCl, 10 mM NaH_2_PO_4_/NaHPO_4_, pH 7.5, 130 mM NaCl, 1% Triton-X-100 and 10 mM NaPPi) in suitable amounts such as to obtain 3 × 10^5^ cells/100 µl. The peptide Ac-DEVD-AMC (20 µM) and 1 ml of Protease Assay Buffer (20 mM HEPES pH 7.5, 10% glycerol, 2 mM DTT) were added to each reaction. The reaction was then incubated at 37 °C for 1 h. The amount of AMC released was measured by the spectrofluorometer (Cary Eclipse Fluorescence Spectrophotometer, Agilent Technologies, Walnut Creek, CA, USA) exciting at 380 nm and detecting in a range between 430–460 nm.

### Phalloidin staining

Haemocytes were isolated 56 h after injection of the vector pIZT/V5-His with or without *Tn*BVank1 as described above and from parasitised larvae. Cells were fixed, permeabilised and blocked as previously described. Subsequently, haemocytes were incubated with TRITC-conjugated phalloidin (Sigma-Aldrich St Louis, MO, USA), diluted 50 µg/ml in 1% BSA for 2 h at room temperature in dark condition. After washing in PBS 1X three times, the slides were mounted with glycerol (Sigma-Aldrich St Louis, MO, USA).

The slides were observed microscopically with Nikon Eclipse 80i equipped with a Nikon Plan Fluor 100x/0.5–1.3 Oil Iris objective and the images were recorded with Nikon Digital Sight DS-U1 camera.

### Hoechst staining

S2 cells (negative control), S2 cells stably expressing *Tn*BVank1 and *H*. *virescens* haemocytes isolated 56 h after injection of the vector pIZT/V5-His with or without *Tn*BVank1 were stained with Hoechst 33258 (Sigma-Aldrich, St Louis, MO, USA) to detect nuclear morphology. Cells were fixed with 4% paraformaldehyde for 10 min, washed with PBS, and stained with 10 *μ*g/mL Hoechst 33258 at room temperature for 10 min in the dark. The cells were washed with PBS 1X three times and the slides were mounted with glycerol for morphologic observation by fluorescence microscopy (NIKON Eclipse 80i) equipped with a Nikon Plan Fluor 40x and 100x/0.5–1.3 Oil Iris objective and the images were recorded with Nikon Digital Sight DS-U1 camera.

### Western blot time course

The *Tn*BVANK1 protein production over time in the polyclonal S2 cell line that stably expresses *Tn*BVank1 was evaluated by western blot analysis. For proteins extraction, S2 cell line expressing *Tn*BVank1, harvested at different successive splitting (2, 4, 10, 20, 26, 39 passages), was resuspended, transferred to sterile 1.5 ml tubes (Eppendorf, Hamburg, Germany) and centrifuged at 1000 *g* for 5 min at room temperature. The cell pellet was then resuspended in lysis buffer (50 mM Tris-HCl, pH 7.8, 150 mM NaCl, 1% Nonidet P-40) to which 1% protease inhibitor cocktail (Sigma-Aldrich, St Louis, MO, USA) was added. After incubation at 37 °C for 10 min, the lysed cells were centrifuged at 16000 *g* for 10 min at room temperature and the supernatant containing soluble proteins was transferred to a new 1.5 ml tube (Eppendorf, Hamburg, Germany). The protein concentration was determined by the Bradford method^52^, using BSA as standard and 100 µg of protein lysate were used for each SDS PAGE gel lane. The viral protein *Tn*BVANK1 was detected using the murine anti-V5 antibody (Life Technologies, Carlsbad, CA, USA) as primary antibody (diluted 1:5000 in 5% milk), and anti-mouse conjugated to horseradish peroxidase (Life Technologies, Carlsbad, CA, USA) (diluted 1:5000 in PBS), as secondary antibody. α-actin was chosen as endogenous control, using the anti- α -actin antibody (Sigma-Aldrich, St Louis, MO, USA) (diluted 1:5000 in 5% milk) and the secondary antibody anti-rabbit conjugated to horseradish peroxidase (Life Technologies, Carlsbad, CA, USA, USA) (diluted 1:15000 in PBS). Finally, the Western blot Chemiluminescent HRP Substrate (LiteAblot, Euroclone, Milan, Italy) to detect target proteins was used.

### Functional proteomics

Functional proteomic experiments were performed on protein lysates of S2 cells stably expressing V-5-tagged *Tn*BVANK1. A total protein extract was subjected to pre-cleaning treatment on underivatised agarose beads and the unbound fraction was immunoprecipitated by incubation onto anti-V5 agarose-conjugated antibody overnight under gentle stirring. An aliquot of the non specific bound material was eluted and used as control.

Beads were collected by centrifugation (3,000 rpm for 5 min) and extensively washed with lysis buffer supplemented with 150 mmol/l NaCl. Elution was performed by competition with V-5 peptide in elution buffer. The presence of *Tn*BVANK1 in the eluted fraction was assessed by Western-blot using anti-V5 antibody.

The eluted proteins were precipitated in methanol/chloroform and then loaded onto a 10% SDS-PAGE. Protein bands were excised, reduced with 10 mM DTT and carboxyamidomethylated with 55 mM iodoacetamide. Tryptic digestion was carried out with 12.5 ng/μl at 4 °C for 2 h in 10 mM NH_4_HCO_3_ buffer, pH 7.8 and then for 16 h at 37 °C. Peptides were then extracted with 10 mM NH_4_HCO_3_ and 1% formic acid in 50% acetonitrile at room temperature. Gel slices from the negative control S2 cells were also analysed.

Peptide mixtures were analysed by LC-MS/MS using a CHIP MS 6520 QTOF equipped with a capillary 1200 HPLC system and a chip cube (Agilent Technologies, Palo Alto, CA, Italy). Peptide analysis was performed using data-dependent acquisition. Mass spectral data were used to search for the UniProt/SwissProt protein database using an in house version of MASCOT (Matrix Science, Boston, USA).

### Coimmunofluorescence analysis

For the immunofluorescent labeling, S2 cells were fixed for 10 min at room temperature in 4% formaldehyde in 1X phosphate buffer saline (PBS) pH 7.5. After permeabilisation for 30 min in 0.1% Triton X-100 solution and washing in PBS 1X, the cells were incubated in 1% BSA for 30 min and, subsequently, with the mixture of two primary antibodies, anti-*Tn*BVANK1^45^ and anti-Alix^53^, 1:200 in BSA 1%, in a humidified chamber at 4 °C overnight. After three washes in PBS 1X, the cells were incubated with the mixture of two secondary antibodies, which were raised in different species (with two different fluorochromes, i.e. TRITC-conjugated against mouse and FITC-conjugated against rabbit) (Sigma-Aldrich St Louis, MO, USA), 1:1000 in 1% BSA for 1 h at room temperature in dark condition. After washing in PBS 1X, the slides were mounted with Fluoroshield^TM^ with DAPI, histology mounting medium (Sigma-Aldrich St Louis, MO, USA).

To measure the total cell fluorescence, the slides were observed microscopically with Nikon Eclipse 80i equipped with a Nikon Plan Fluor 100x/0.5–1.3 Oil Iris objective and the images were recorded with Nikon Digital Sight DS-U1 camera. The intensity of the cell fluorescence was measured using the ImageJ software (http://rsbweb.nih.gov/ij/).

### *In vivo* and *in vitro* Alix silencing by RNA interference

RNA interference (RNAi) experiments both *in vivo* and *in vitro* were performed using double-stranded (ds)RNA of the Alix gene (Accession Number Q9VB05) (see Supplementary Fig. S6). As negative control dsRNA of the *Apis*OBP3 gene (Accession Number 001160057), which is not expressed in S2 cells and in *H*. *virescens* haemocytes, was used.

Part of the coding sequences for Alix and *Apis*OBP3 proteins were amplified by PCR with specific primers containing the T7 binding site at their 5′ ends (underlined):

S2AlixF 5′-TAATACGACTCACTATAGGGAGAATTGGCGAGGAGATTGCT-3′

S2AlixR 5′-TAATACGACTCACTATAGGGAGAATCGCGAAGTCATCACCAAT-3′

*H*.*vir*AlixF 5′-TAATACGACTCACTATAGGGAGATGAAGGTATGGAGATACTGA-3′

*H*.*vir*AlixR 5′-TAATACGACTCACTATAGGGAGAATCGAAGGTGTAGCAGCTG-3′

OBP3F 5′-TAATACGACTCACTATAGGGGTAATAAAACAAGGCGCACAG-3′

OBP3R 5′-TAATACGACTCACTATAGGGATCGTCGTCGGATCAAGGAA-3′

PCR products were gel purified (Quantum Prep™ Freeze’N Squeeze DNA Gel Extraction Spin Columns, Biorad, Hercules, CA, USA) and Alix dsRNA and *Apis*OBP3 dsRNA were prepared using the RNAi MEGAscript^®^ kit (Ambion, Austin, TX, USA) following the manufacturer’s instructions. The quality of dsRNA obtained was observed on an agarose gel and the quantity was measured using a NanoDrop (ND-1000, Thermo Scientific, Waltham, MA, USA).

For *in vitro* RNAi, dsRNAs (16 µg for each well) were directly added to the culture medium. A number of cells equal to 4 × 10^6^ cells in 2 ml were plated 24 h before silencing in a 6-well plate. Wells contained S2 cells and S2 cells stably expressing *Tn*BVank1, respectively. The same procedure was performed in control cells, but no dsRNA was added. After 2 hours the medium containing the dsRNA was replaced with fresh medium without dsRNA and at different time points (T0, T6 and T12 h) 2 × 10^6^ cells were harvested for RNA extraction and 3.5 × 10^5^ cells for the post silencing caspase-3 assay.

For *in vivo* RNAi, 0.5 µg of both dsRNAs were mixed with 0.5 µg of pIZT/V5-His-ANK1 and 3 µl of transfection reagent (Lipofectin) and incubated for 20 min at room temperature before injection into the haemocoel of fifth instar *H*. *virescens* larvae (day one). Treated larvae were processed for qPCR analysis 56 h after the dsRNA administration.

### Post silencing Alix and *Tn*BVank1 expression in S2 cells

In order to verify the Alix silencing, its transcript expression level was detected by qPCR and, on the same samples, the *Tn*BVank1 transcript level expression was determined. RNA was extracted at different time points from cells (T0, T6 and T10 h after adding dsRNA) using TRI Reagent (Sigma-Aldrich St Louis, MO, USA) and RNA quality was evaluated on an agarose gel. For qPCR, 1 µg of total RNA was treated with DNase (Deoxyribonuclease I Amplification Grade, Life Technologies, Carlsbad, CA, USA) and then it was reverse transcripted using Super Script ™ ІІІ First-Strand Synthesis System for RT-PCR (Life Technologies, Carlsbad, CA, USA, USA), with oligo-dT primers, following the manufacturer’s protocol. qPCR was performed using GoTaq^®^ qPCR Master Mix (Promega, Fitchburg, WI, USA) on an ABI Prism^®^ 7500 Fast Real-Time PCR System Thermal Cycler (Applied Biosystem, USA). Sequence specific primers, each in a concentration of 0.3 µM were:

AlixF 5′-CGCCCTGCAGAGCAACA-3′

AlixR 5′-AGGGCACTGCCGCTGG-3′

Ank1F 5′-ATCATGAGGGGCATGCATG-3′

Ank1R 5′-AGCACCAGCGCTGTGAAGT-3′

As a reference gene, the gene coding for the *Drosophila* ribosomal protein L32 (RPL32, Accession Number P04359)^54^ was used. Primers were:

Rpl32f 5′-TGCTAAGCTGTCGCACAAAT-3′

Rpl32r 5′-GTTCGATCCGTAACCGATG-3′

All qPCR analyses were carried out in triplicate. Quantification analysis of amplification was done using the comparative C_T_ (ΔC_T_) method. The efficiencies of the two amplicons were approximately equal (Alix = 0.744509; Ank1 = 0.902369; Rpl32r = 0.841033).

### Post silencing Alix and *Tn*BVank1 expression in haemocytes

In order to verify the Alix silencing, its transcript expression level was detected by qPCR also in haemocytes and on the same samples the *Tn*BVank1 transcript level expression was determined. RNA was extracted at 56 h after injection using TRI Reagent (Sigma-Aldrich St Louis, MO, USA), the quality and quantity were determined using NanoDrop spectrophotometer (ND-1000, Thermo Scientific, Waltham, MA, USA) and potential DNA contamination was eliminated with DNase (Deoxyribonuclease I Amplification Grade, Life Technologies, Carlsbad, CA, USA). cDNA was obtained as described above. qPCR was performed using GoTaq^®^ qPCR Master Mix (Promega, Fitchburg, WI, USA) in a ABI Prism^®^ 7500 Fast Real-Time PCR System Thermal Cycler (Applied Biosystem, USA). Sequence specific primers, each in a concentration of 0.3 µM, were:

AlixF 5′-GTTGAACATCTTGGCACGGT-3′

AlixR 5′-CAAAGCATGGACTCACGAGC-3′

*Tn*BVank1 and reference gene primer sequences are reported above. All qPCR analyses were carried out in triplicate. Quantification analysis of amplification was done using the comparative C_T_ (ΔC_T_) method. The efficiencies of the three amplicons were approximately equal (Alix = 0.931310; Ank1 = 0.898875; EF1α = 0.874059; Rp13 = 0.894415).

### Statistical analysis

All data were presented as mean ± SD of three independent biological replicates and were compared with analysis of variance (ANOVA) and Tukey’s test using GraphPad Prism 6 software, La Jolla, California, USA.

## Electronic supplementary material

Supplementary Information
