# Supplementary material for: The multifunctional polydnavirus TnBVANK1 protein: impact on host apoptotic pathway
Source: Sci Rep. 2017 Sep 18;7:11775. doi: 10.1038/s41598-017-11939-x (PMC5603617; doi:10.1038/s41598-017-11939-x)
Supplement: Supplementary file 1 — Supplementary Information [file 41598_2017_11939_MOESM1_ESM.pdf]

The multifunctional polydnavirus *Tn*BVANK1 protein: impact on host apoptotic pathway

Rosanna Salvia,[a] Gerarda Grossi,[a] Angela Amoresano,[b] Carmen Scieuzo,[a] Marisa Nardiello,[a] Chiara Giangrande,[b] Ilaria Laurenzana,[c] Vitalba Ruggieri,[c] Sabino A. Bufo,[a] S. Bradleigh Vinson,[d] Monica Carmosino,[a] David Neunemann,[e] Heiko Vogel,[e] Pietro Pucci,[b] Patrizia Falabella[a*]

[a] Department of Sciences, University of Basilicata, Via dell'Ateneo Lucano 10, 85100 Potenza, Italy.

[b] Department of Chemical Sciences, Federico II University, Via Cintia 6, 80126 Naples, Italy.

[c] Laboratory of Preclinical and Translational Research, IRCCS Referral Cancer Center of Basilicata (CROB), Via Padre Pio 1, 85028 Rionero in Vulture (PZ), Italy.

[d] Department of Entomology, Texas A&M University, 370 Olsen Blvd, College Station, TX 778432475, USA.

[e] Department of Entomology, Max Planck Institute for Chemical Ecology, Hans-Knöll-Straße 8, D07745 Jena, Germany.

[*] Corresponding author:

Patrizia Falabella, Department of Sciences, University of Basilicata, Via dell'Ateneo Lucano 10, 85100 Potenza, Italy.

**Band**	**MW**	**Protein**	**Drosophila Melanogaster**	**Homo sapiens**	**Score**	**N peptides**	**Molecular weight**
**1**	>180 kDa	**P29742**	Clathrin heavy chain (*chc*)	Q00610	278	6	192937
		**Q7JV23**	Acetyl CoA carboxylase (*ACC*)	Q13085	69	2	263010
**2**	100-180 kDa	**A1ZBE9**	Metionyl tRNA synthase (*MetRS*)	P56192	73	3	113269
		**Q9VS57**	Sec63 translocase (*Sec63*)	Q9UGP8	64	2	86483
**3**	70-100 kDa	**Q9VB05**	ALG-2 interacting protein X (*ALIX*)	Q8WUM4	99	2	92938
		**Q94527**	Nuclear factor NF-kappa-B p110 subunit *(Rel)*	Q00653	75	2	97894
		**Q8T3L1**	Acyl-CoA synth long-chain (*Acsl*)	O95573	41	3	81296
		**Q24156**	Transcription factor (*stwl*)	Q14978	39	2	113129
**4**	60-70 kDa	**Q9VA69**	Prolyl-4-hydroxylase-alpha EFB (*PH4alphaEFB*)	P13674	196	4	63360
		**Q9VHN7**	Putative: transketolase	P29401	105	2	68673
		**P21187**	Polyadenylate-binding protein (*pAbp*)	Q13310	86	2	66074
**5**	55-60 kDa	**Q8SXQ1**	Putative: Alpha-aminoadipic semialdehyde	P49419	164	4	58734
		**O62619**	Piruvate kinase (*Pyk*)	P14618	144	5	57950
		**P54399**	Protein disulfide isomerase (*Pdi*)	P07237	123	2	56031
		**Q7KW39**	Probable: methylmalonate-semialdehyde dehydrogenase	Q02252	60	2	59740

		**Q03017**	NF-kappa-B inhibitor cactus	P25963	113	4	53818
**7**	50 kDa	**O77466**	Thiolase (*Thiolase*)	P55084	95	3	50987
**9**	45-40 kDa	**P50887**	Ribosomal protein L22 (*RpL22*)	P35268	120	3	32311
**10**	40-35 kDa	**P19889**	60S ribosomal protein P0 (*RpLP0*)	P05388	169	3	34295
		**Q9V3Y4**	Mitochondrial carrier homolog 1 (*Mtch*)	Q9Y6C9	123	2	35217
		**Q8MRY4**	Putative: double strand RNA binding (*blanks*)	P78563	46	2	34982
**12**	30 kDa	**Q9V447**	Protein Kr-h2 (*Kr-h2*)	P57088	61	2	31309
**13**	25 kDa	**A1Z7Z4**	CG1648-PB, isoform B	?????	204	5	23839
		**Q24186**	40S ribosomal protein S5 (*RpS5a*)	P46782	130	4	25760
		**Q8MLY8**	40S ribosomal protein S8 (*RpS8*)	P62241	77	2	23859
**14**	25 kDa	**P02255**	Histone 1 (*His1*)	P07305	47	2	26637
**15**	22-25 kDa	**Q9V3P0**	Peroxiredoxin 1 (*Jafrac1*)	P32119	146	4	21952
		**Q9VZ23**	GTP-binding nuclear protein Ran (*Ran*)	P62826	124	4	24891
		**O18332**	FI01544p (*Rab1*)	P62820	122	3	23034
		**Q9VBU6**	CG11857-PA	O15258	121	2	23877
		**Q9VA91**	40S ribosomal protein S7(*RpS7*)	P62081	116	3	22156
		**P20432**	Glutathione S-transferase D1 (*GstD1*)	P30711	45	2	22710
**16**	22 kDa	**P41093**	60S ribosomal protein L18a (*RpL18A*)	Q02543	39	2	21073
**17**		**Q4QQB9**	LP21121p		52	2	20694
**19**		**O97471**	Microsomal glutathione S-transferase-like protein (*Mgstl*)	P10620	93	2	16842
		**P39018**	Ribosomal protein S19a	P39019	71	3	17394

			(*RpS19a*)				
**20**	15 kDa	**Q9VJ19**	Ribosomal protein L30 (*RpL30*)	P62888	128	2	12398
		**Q9VIQ8**	Cytochrome c oxidase subunit 4 isoform 2, (*COX4*)	Q96KJ9	66	2	20678
		**P48149**	Ribosomal protein S15a (*RpS15Aa*)	P62244	57	2	14933
		**P55828**	Ribosomal protein S20 (*RpS20*)	P60866	53	2	13593
		**Q9W237**	Ribosomal protein S16 (*RpS16*)	P62249	50	2	16878

Table S1. List of *Tn*BVANK1 protein interactors obtained by coimmunoprecipitation experiments. Proteins were annotated against the genome of *Drosophila melanogaster* and then compared to *Homo sapiens* genome. For each protein the score, the number of unique peptide and the molecular weight are reported.

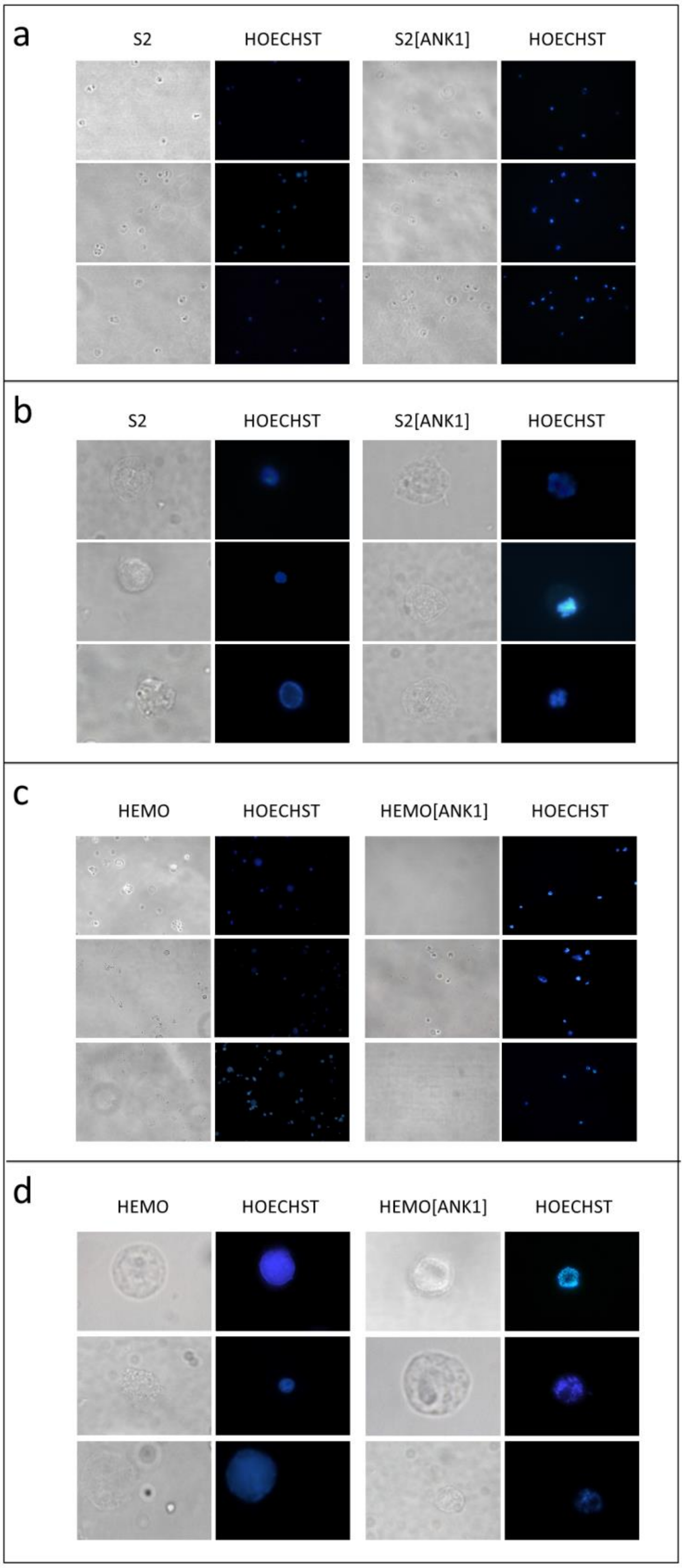
a
S2
HOECHST
S2[ANK1]
HOECHST
b
S2
HOECHST
S2[ANK1]
HOECHST
c
HEMO
HOECHST
HEMO[ANK1]
HOECHST
d
HEMO
HOECHST
HEMO[ANK1]
HOECHST

Figure S1. Effect of *Tn*BvAnk1 on the morphology of the haemocytes and S2 cells nuclei. Nuclei were detected with Hoechst 33258 staining in S2 control (S2) and polyclonal S2 cells (S2 [ANK1]) at magnification 40X (a) and 100X (b) and in heamocytes control (Hemo) and in haemocytes *in vivo* transfected with *Tn*BvAnk1 (Hemo[ANK1]) at magnification 40X (c) and 100X (d). Images were generated with immunofluorescence microscopy using Nikon Eclipse 80 and recorded with Nikon Digital Sight DS-U1 camera and ImageJ software.

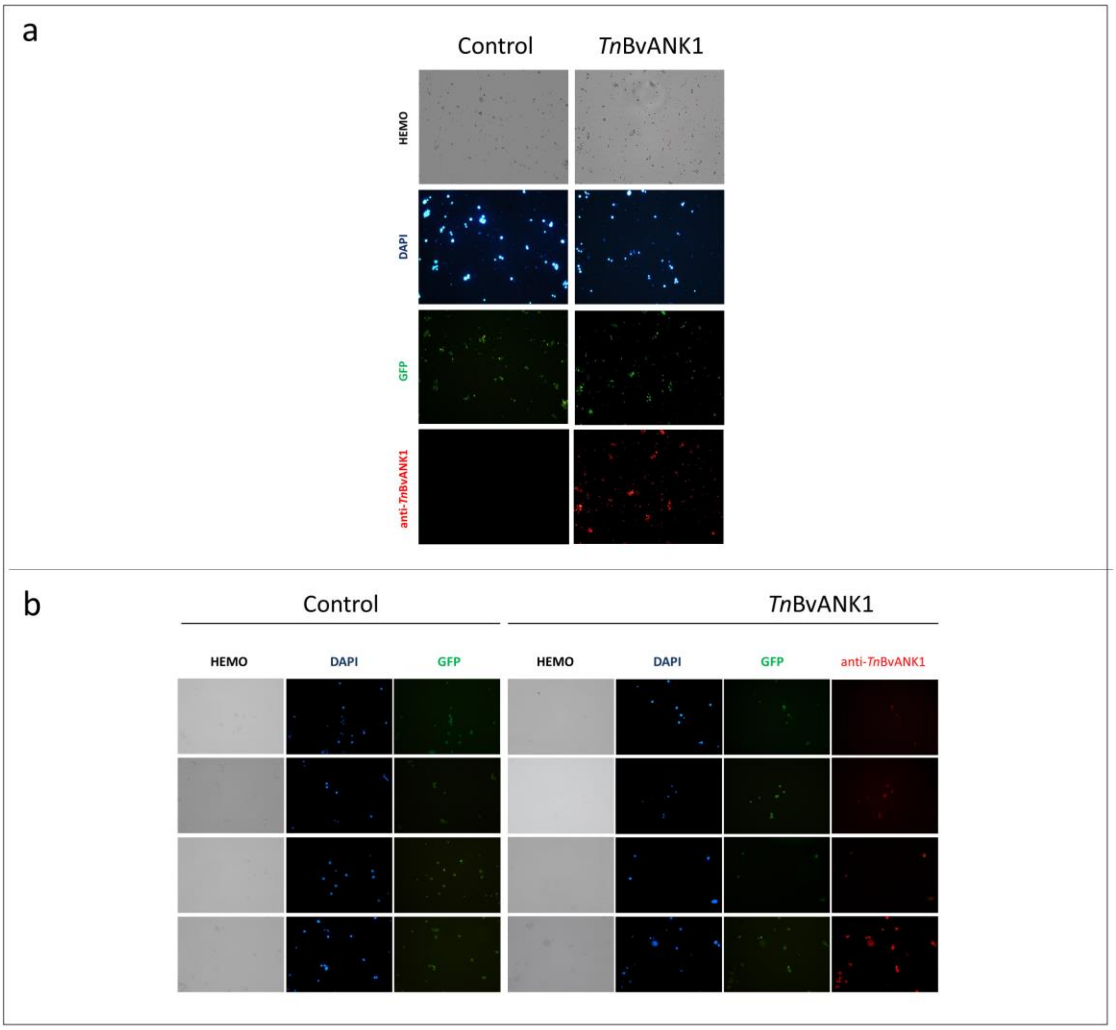

Figure S2. Efficiency of *in vivo* transfection. Immunofluorescence of *in vivo* transfected haemocytes showed epifluorescent (for GFP) signal in both control (haemocytes transfected with the empty vector) and *Tn*BVANK1 (haemocytes transfected with *Tn*BVank1) (green signal). Red signal of *Tn*BVANK1 was detected for haemocytes transfected with *Tn*BVank1 (ANK1). Images were generated with immunofluorescence microscopy using Nikon Eclipse 80i at magnification 20X (**a**) and 40X (**b**), the images were recorded with Nikon Digital Sight DS-U1 camera and ImageJ software.

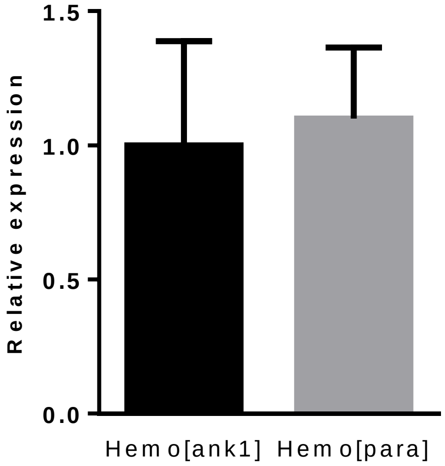

Figure S3. *Tn*BVank1 transcript levels in haemocytes after parasitism and after *in vivo* transfection. Expression level of *Tn*BVank1 transcripts in *H. virescens* haemocytes 56 h after *in vivo* transfection (Hemo[ank1]) and after parasitisation (Hemo[para]), normalised to the endogenous controls (EF1α and RP13[49]).

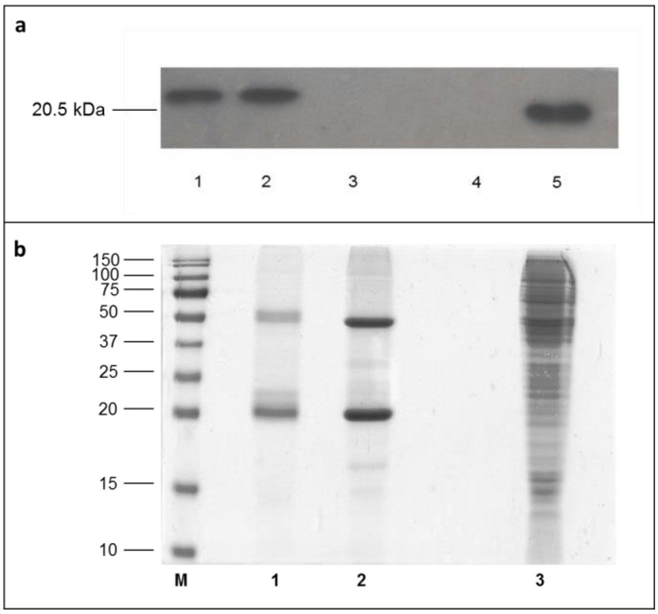

Figure S4. Western blot and SDS PAGE post coimmunoprecipitation on polyclonal S2 cell lines protein extract. a) Western blot post- immunoprecipitation on polyclonal S2 cells stably expressing *Tn*BVank1 and S2 cells (control). The loaded samples were: 1) total extract; 2) unbound precleaning; 3) unbound V5; 4) control elution; 5) sample elution. Detection of *Tn*BVANK1 was determined by using anti-V5 antibody. **b)** SDS PAGE post-coimmunoprecipitation on protein extract from polyclonal S2 cells stably expressing *Tn*BVank1 and S2 cells (control). The immunoprecipitated complexes were fractionated by SDS-PAGE at 12% and the gel was stained with Coomassie blue. The loaded samples were: M) protein molecular weight marker; 1) control elution; 2) sample elution; 3) total extract. Spots were extracted from the sample and control elution lanes in order to obtain peptides to be analysed with mass spectrometry LC-MS/MS.

>H. Virescens 60S RIBOSOMAL PROTEIN L3

ATGACAGCTCACCACTGAAATAAAATCGTTTTCAATCGGCCATTTTTGCCGCGACTGTCTTCGAACATGTCGCATAGAAA
GTTTTCGGCGCCCCGTCATGGGTCCATGGGATTCTACCCCAAGAAGAGGTCCCGCCGTCATCGTGGTAAGGTGAAGGCAT
TCCCCAAGGATGATGCCAGCAAACCTGTCCACCTTACCGCCTTCATTGGTTACAAGGCCGGTATGACCCACGTGGTTCGT
GAACCTGACCGTCCTGGATCAAAGATCAACAAGAAGGAGATCGTTGAGGCTGTGACTATCATCGAGACGCCACCCATGGT
GTGCGTTGGTGTGGTTGGTTACATCGAAACTCCTCATGGTCTCCGCGCTCTGCTCACTGTCTGGGCTGAGCACATGTCCG
AGGACTGCCGCCGTCGCTTCTACAAGAACTGGTACAAATGCAAGAAGAAGGCATTCACAAAATCCAGCAAGAAATGGCAG
GATGAGCTTGGACGCAAGTCCATTGAGAAGGACTTCAAGAAAATGATCCGCTACTGCAGTGTGATCAGGGTTATTGCCCA
CACCCAGATGAAGTTGCTGAAGCAGCGTCAAAAGAAAGCTCACATTATGGAGATCCAAGTCAACGGTGGATCCATTGAAG
ACAAAGTAAAATGGGCAAGAGAACATCTTGAGAAGCCCATTCCCATTGACTCTGTGTTTGCCCAAGATGAGATGATTGAT
TGCATTGGCGTCACCAAGGGTAAAGGATACAAGGGTGTGACCTCCCGTTGGCACACAAAGAAGTTGCCTCGCAAGACACA
CAAGGGTCTGCGTAAAGTTGCTTGTATTGGAGCGTGGCATCCTTCAAGAGTTTCATTCACTGTTGCCCGTGCTGGTCAGA
AGGGCTACCACCATCGTACTGAGATGAACAAGAAGATCTACC

>H. virescens ELONGATION FACTOR 1-ALPHA

GCTCTTCCGATCTCTCGGATATTACACGTGGATTGTAATCCGTGACTAACCAAAAATGGGCAAGGAAAAGATTCATATTA
ACATTGTCGTCATTGGACACGTCGACTCCGGCAAGTCCACAACCACCGGTCACTTGATCTACAAATGCGGTGGTATCGAC
AAACGTACCATCGAGAAGTTCGAGAAGGAGGCCCAGGAAATGGGTAAGGGTTCCTTCAAATACGCCTGGGTATTGGACAA
ACTGAAGGCTGAGCGTGAACGTGGTATCACCATCGATATCGCCCTGTGGAAGTTCGAAACCGCCAAATACTATGTCACCA
TCATCGACGCTCCCGGACACAGAGATTTCATCAAGAACATGATCACTGGAACTTCCCAGGCTGACTGCGCCGTGCTCATC
GTCGCCGCTGGTACCGGTGAGTTCGAGGCTGGTATCTCCAAGAACGGACAGACCCGTGAGCACGCTCTGCTCGCCTTCAC
CCTCGGAGTCAAGCAGCTGATTGTGGGCGTCAACAAAATGGACTCCACTGAGCCCCCATACAGCGAATCCCGTTTCGAGG
AAATCAAGAAGGAAGTATCTTCCTACATCAAGAAGATCGGTTACAACCCAGCTGCCGTCGCTTTCGTACCCATTTCTGGC
TGGCACGGAGACAACATGTTGGAGGCGTCAACCAAAATGCCCTGGTTCAAGGGATGGAACGTCGAGCGCAAGGAGGGTAA
GGCTGAAGGTAAATGCCTCATTGAGGCCCTTGACGCCATCCTGCCCCCTGCTCGTCCCACAGACAAGGCCCTGCGTCTTC
CCCTCCAGGACGTATACAAAATCGGTGGTATCGGTACGGTGCCCGTAGGCAGAGTCGAAACTGGTATCTTGAAGCCTGGT
ACCATCGTCGTCTTCGCCCCCGCCAACATCACCACTGAAGTCAAGTCTGTGGAGATGCACCACGAAGCTCTCCAAGAGGC
CGTACCTGGTGACAACGTTGGTTTCAACGTAAAGAACGTTTCCGTCAAGGAGTTGCGTCGTGGTTACGTCGCTGGTGACT
CCAAGAACAACCCACCCAAGGGCGCCGCCGATTTCACAGCACAGGTCATCGTGCTCAACCACCCTGGTCAAATCTCAAAC
GGATACACACCCGTGTTGGATTGCCACACAGCTCACATTGCCTGCAAGTTCGCCGAAATCAAAGAGAAGGTTGACCGTCG
TACTGGTAAATCCACTGAAGACAACCCTAAGTCCATCAAGTCTGGTGACGCCGCCATCGTCAACCTGGTTCCCTCCAAGC
CTCTGTGTGTTGAGTCCTTCCAGGAATTCCCTCCCCTTGGTCGTTTCGCCGTGCGTGACATGAGGCAGACGGTCGCTGTG
GGTGTCATCAAGGCAGTGAACTTCAAGGAAGTTGGTGGCGGTAAGGTGACCAAAGCCGCCGAGAAGGCCACCAAGGGCAA
GAAGTAGCTAGCGCTGTTAACAGCACAATTTTTCATTCAACTGCGATACTTCATTCACCGCAAGGTGTTCCGAAGGAAAG
AAGGGCTACAAACTCATTCCTTTTCTATATTTTTTACAAGGCTTATACTGTAACATTATTTTATAATTTATA
TAAGGTTATATCTGAACTATTTTGTTATAACTGCAAACTAATGTGTAAATTCATAGAATAAAGGTACAGTATA
A

Figure S5. Nucleic sequences of *H. virescens* 60S Ribosomal Protein L3 (RPL3) and Elongation Factor 1-Alpha (EF1α). Nucleotide sequences of reference genes used for the qPCR performed after the Alix silencing in *H. virescens* haemocytes are retrieved from in-house transcriptome databases.

```
D. melanogaster     --------------MSKFLGVPLKKPSEVDVIKPLNNLIQSTYNGASEEEKGKYGEAVNE
H. virescens        MRLICSLSYSFVEMAAELLFVPFKKSSDVDIVKPLRNLINSTYNTGDH--HEDYTEALNE
                                   :::* **:** *:**::***.***:**** ...  : .* **:**

D. melanogaster     FSKQRNTAIWKFFEKYEASLEIVYAYYDQICALETKISVSELQIPFKWKDAFDKGSIFGG
H. virescens        LSRLRANAIWKVFEK--TSLDIIYNYHDQLASLESKVPPQEVQIPFKWKDAFDKGSLFGG
                    :*: * .****.***  :**:*:* *:**:.:**:*:  .*:**************:***

D. melanogaster     KISLTHTSLLYEKVCVLFNIAALQSNIAANQSLDSDDGLKLTIKLLQQSAGIFQYLKGAT
H. virescens        RMSLTISSLAYERMCILFNIAAMQSLIASQQPVETEESLKQAAKLFQQAAGVFLYLKANI
                    ::*** :** **::*:******:** **::* :::::.** : **:**:**:* ***.

D. melanogaster     PAAVPSEPTPDLSQDTLTVLQALMVAQAQEVFILKAIKDNLKDQIIAKLCCQAEESYADV
```

```
H. virescens        MMAVHQETTPDLHPETLDALAKLMLAQAQEVIAFKCIRDEMKDSMVAKVCAQCDELYTDA
                      ** .* ****  :** .*  **:******: :*.*:*::**.::**:*.*.:* *:*.

D. melanogaster     LRAMQKESVRSLWEKEWIPTIAGKQAGFHALTQLYQSLVCRAAKKIGEEIARLRNAIDLF
H. virescens        LRAMQKEQLKSLWERDWLPVMTSKQQAFRGLAQLYQAQVCRASKSVGEEIARLALADELL
                    *******.::****::*:*.::.** .*:.*:****: ****:*.:*******  * :*:

D. melanogaster     KAAQTRSGNETYLDEYFSRAKRNLTESTKDNEFIYNEIIPELSTLTSPGKAQLAKPLPIA
H. virescens        RGAARGVAPAAWLGEQQARAARALAAARRDNDFIYHERVPDAAALEPLARAAVAKPAEPP
                    :.*     .  ::* *  :** * *: : :**:***.* :*: ::*   .:* :***

D. melanogaster     VPLAENFKDIFSSLVPVELHRALTASDMRRNEIVNVEIMKLREATQTLNAVLASLNLPAA
H. virescens        AR-WAAARDLFAALVPHAVHAALQAAAARRADLVAREVAALRDATQLLNAVLAELSLPAC
        .                  :*:*::***  :* ** *:  ** ::*  *:  **:*** ******.*.***.

D. melanogaster     VETADGNSGLPPSLKEKANEVRQKGGIENVQTMLKDLPELLNRNREILDETERLLDEERD
H. virescens        LEGA-GAGALPDSIRARAQAVRDAGGLPELTRLMAELPELLQRNRDILDEAERMLREEAE
                    :* * * ..** *:: :*: **: **: ::  :: :*****:***:****:**:* ** :

D. melanogaster     SDNQLRAQFKDRWTRISSDKLTEMFRTNAKKYREVITNAIEADKVVRQKFEANQKGIQLL
H. virescens        ADSALRQQFGARWARTESAKLTDAFRANADKYRQIIDNAVRADAIVQQKLAQHRDNIALL
                    :*. ** **  **:* .* ***: **:**.***::* **:.** :*:**:  .:. * **

D. melanogaster     SLPPDQIQQSLPSASGSVDPNC---SSVQRLKKLMDDVETIKAEREAIESELKGATFNMK
H. virescens        GGSEQELSAGVPDGPARDQPDAGAPDAVRRLRQLCADVEALKAERDAIEAELKDTTVDLR
                    .   :::. .:*.. .  :*:.   .:*:**::*  ***::****:***:*** :*.:::

D. melanogaster     DEFLIALQKDGAIDEPALSLARIGQVLNPLQQQVRESVERQQSLVSEIQSAHGAFVSETG
H. virescens        ERFLAALAADGCVDEPALSAGALGSALAPLQRRAAATLARQEELLAAVQAAHGALTAASR
                    :.** **  **.:****** . :*..* ***::.  :: **:.*:: :*:****:.: :

D. melanogaster     S------------------CGSSRDTLYQELATAFDSYIELSGNLQEGTKFYNDLTQLLV
H. virescens        PDAPXRDQPDAGALTAARGGASGRDAALGRLAAAADAFQELTANLNEGIKFYNDLTQLLV
                                        .*.**:   .**:* *:: **:.**:** ***********

D. melanogaster     VFQNKISDFVFARKTEKEELLKDLTTESSRQACPATPALPSHYASTSGSGSDI-------
H. virescens        AFQNKVSDFCFARKTEKDELLKDLTQEASRGSVRPAPSPPQHHAAAAEPSARREPPPRPP
                    .****:*** *******:******* *:** :   :*: *.*:*:::  .:

D. melanogaster     ----PPGSAP---SVPPAASTANIPYPAQVQGMPIPYGAQPGVPYPAYVPAPMPQSFNPY
H. virescens        PPAAAPGPAPAPAPAPAAAASASLPYPQQPQGMPLPYGAGAAYPY---YGAPVPQLYNPY
                         ** **    .* **::*.:*** * ****:****  . **     **:** :***

D. melanogaster     ATLPYPGNYQYQG---------------FPQGPPPGHYGTYPGSYANQQGGYPNQKPPGW
H. virescens        ATLPYPHHAPRMPPPQPYQPYQYPAPAPFAPQPPPAGYNPYPPQ----------------
                    ****** .                    *   ***. *  ** .
```

Figure S6. Multiple alignment of *H. Virescens* Alix protein sequence with the homolog from *D. melanogaster* (Q9VB05). The alignment shows identity equal to 46% and query cover equal to 97%.

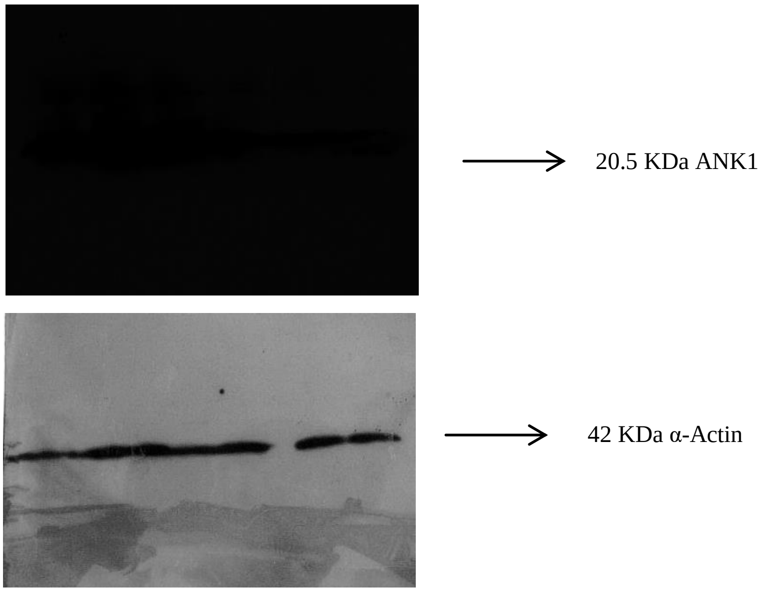

Figure S7. Original Western blot image for Fig. 2

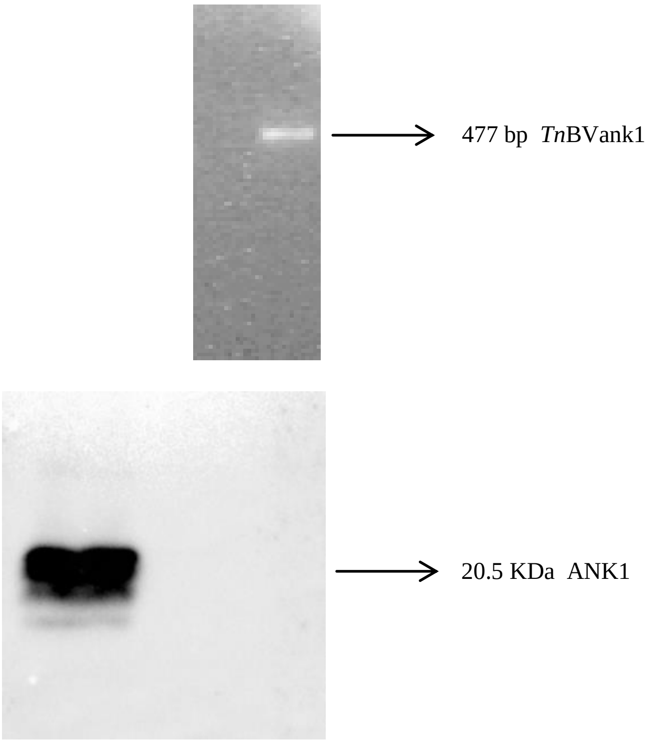

Figure S8. Original gel and Western blot images for Fig. 3.

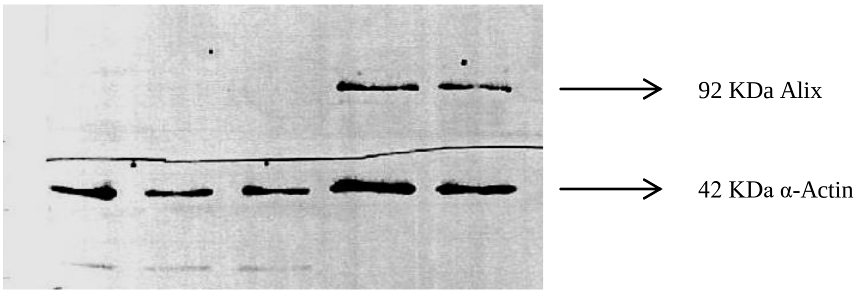

Figure S9. Original Western blot image for Fig. 6b. After the transfer on a nitrocellulose membrane, the membrane was cut at the level of 60KDa and the two stripes were incubated separately with the primary antibodies (anti-Alix and anti- α-Actin) and with the secondary antibodies (anti-mouse and anti-rabbit, both conjugated to horseradish peroxidase). Then, the two pieces were combined and proteins of interest were detected together by Western blot Chemiluminescent HRP Substrate. To have a better order of the samples, the membrane was turned upside down, as reported in Fig. 6b.

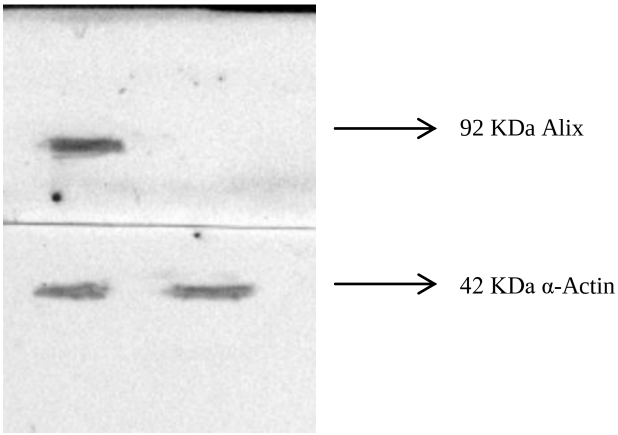

Figure S10. Original Western blot image for Fig. 7b. After the transfer on a nitrocellulose membrane, the membrane was cut at the level of 60KDa and the two stripes were incubated separately with the primary antibodies (anti-Alix and anti- α-Actin) and with the secondary antibodies (anti-mouse and anti-rabbit, both conjugated to horseradish peroxidase). Then, the two pieces were combined and proteins of interest were detected together by Western blot Chemiluminescent HRP Substrate.
